# Synthesis and Characterization of Iridium(III) Complexes with Substituted Phenylimidazo(4,5-*f*)1,10-phenanthroline Ancillary Ligands and Their Application in LEC Devices

**DOI:** 10.3390/molecules29010053

**Published:** 2023-12-21

**Authors:** Bárbara Vásquez, Max Bayas, Paulina Dreyse, Juan Luis Palma, Alan R. Cabrera, Elena Rossin, Mirco Natali, Cesar Saldias, Iván González-Pavez

**Affiliations:** 1 Departamento de Química Física, Facultad de Química y de Farmacia, Pontifica Universidad Católica de Chile, Av. Vicuña Mackenna 4860, Macul 7820436, Chile; buvasquez@uc.cl; 2 Departamento de Química, Facultad de Ciencias Naturales, Matemática y del Medio Ambiente, Universidad Tecnológica Metropolitana, Las Palmeras 3360, Ñuñoa, Santiago 7800003, Chile; 3 Departamento de Química Inorgánica, Facultad de Química y de Farmacia, Pontificia Universidad Católica de Chile, Av. Vicuña Mackenna 4860, Macul 7820436, Chilearcabrer@uc.cl (A.R.C.); 4 Department of Chemistry, Faculty of Science, Universidad de Chile, Las Palmeras 3425, Ñuñoa, Santiago 7800003, Chile; 5 Engineering School, Universidad Central de Chile, Santa Isabel 1186, Santiago 8330601, Chile; 6 Center for the Development of Nanoscience and Nanotechnology (CEDENNA), Santiago 9170124, Chile; 7 Dipartimento di Scienze Chimiche, Farmaceutiche ed Agrarie, Università degli Studi di Ferrara, Via L. Borsari 46, 44121 Ferrara, Italyntlmrc@unife.it (M.N.); 8 Dipartimento di Scienze Chimiche, Università degli Studi di Padova, Via F. Marzolo 1, 35131 Padova, Italy

**Keywords:** iridium, LEC, phenylimidazo(4,5-*f*)1,10-phenanthroline, photophysical

## Abstract

In this work, we report on the synthesis and characterization of six new iridium(III) complexes of the type [Ir(C^N)_2_(N^N)]^+^ using 2-phenylpyridine (**C1**–**3**) and its fluorinated derivative (**C4**–**6**) as cyclometalating ligands (C^N) and R-phenylimidazo(4,5-*f*)1,10-phenanthroline (R = H, CH_3_, F) as the ancillary ligand (N^N). These luminescent complexes have been fully characterized through optical and electrochemical studies. In solution, the **C4**–**6** series exhibits quantum yields (Ф) twice as high as the **C1**–**3** series, exceeding 60% in dichloromethane and where ^3^MLCT/^3^LLCT and ^3^LC emissions participate in the phenomenon. These complexes were employed in the active layer of light-emitting electrochemical cells (LECs). Device performance of maximum luminance values of up to 21.7 Lx at 14.7 V were observed for the **C2** complex and long lifetimes for the **C1**–**3** series. These values are counterintuitive to the quantum yields observed in solution. Thus, we established that the rigidity of the system and the structure of the solid matrix dramatically affect the electronic properties of the complex. This research contributes to understanding the effects of the modifications in the ancillary and cyclometalating ligands, the photophysics of the complexes, and their performance in LEC devices.

## 1. Introduction

Ionic transition metal complexes (iTMCs) based on iridium(III) have been widely used in OLED (Organic Light Emitting Diodes) or LEC (Light Emitting Electrochemical Cells) devices due to their attractive photochemical and photophysical properties [1,2,3,4]. These complexes present a strong ligand field and an efficient spin-orbital coupling (SOC) leading to high emission quantum yields and long triplet excited state lifetimes [4].

Regarding electroluminescent devices, OLEDs possess an intricate multilayer structure and require elaborate processing methods, rendering them challenging to produce at lower costs [5,6,7]. Due to these limitations, LECs could be one of the most promising lighting devices in terms of architectural simplicity, which leads to reduced production costs and enhanced processability, thereby fostering rapid development. In their simplest configuration, these devices comprise a single active layer placed between a transparent anode and an air-stable cathode. In this context, significant results have been obtained using iTMC as active layer [3,8]. Another advantage of LEC active components is their solubility in common solvents [9], which allows their application at the anode by simple techniques such as spin-coating. Due to the high solubility range, various types of ionic additives, such as polyelectrolytes (PI) or ionic liquids (IL), can be also incorporated to improve device performance due to ionic mobility [10,11].

The most common complexes employed in LEC devices are based on [Ir(C^N)_2_(N^N)]^+^, where C^N is a cyclometalating ligand, such as 2-phenylpyridine (ppy), and N^N is an ancillary ligand, such as bipyridine (bpy) or 1,10-phenanthroline (phen) derivatives. Due to the localization of the frontier orbitals onto these ligands, structural variations play a significant role in color tuning and emission efficiency in optoelectronic devices [12]. In general, the ligands can be modified with the purpose of stabilizing or destabilizing the frontier orbitals, changing the HOMO-LUMO energy gap (H-L gap), and therefore the different electronic properties and emission energy. For example, the use of N^N ligands with high rigidity and electron delocalization, such as ligands derived from phenylimidazo(4,5-*f*)-1,10-phenanthroline, affects the LUMO energy due to an increase in the acceptor π* character [13,14,15]. Ligands with electron-donating substituents increase the LUMO energy, while electron-withdrawing substituents have the opposite effect [16,17,18]. In this way, complexes with N^N ligands derived from 2-phenyl-1H-imidazo-(4,5-*f*)-1,10-phenanthroline exhibit high electron delocalization, showing high emissions and longer excited state lifetimes, a sought-after feature for electroluminescent devices [19,20].

Although numerous reports of LECs have displayed performance in terms of efficiency, stability, and brightness [21,22], the challenge persists in identifying systems that effectively integrate these parameters. Based on the above and to explore solid-state lighting with a simple system based on Ir-iTMC, this work presents the development of six new LEC devices based on Ir(III) complexes using cyclometalated ligands derived from 2-phenylpyridine and ancillary ligands derived from 1H-imidazo-(4,5-*f*)-1,10-phenanthroline. The properties of these complexes allow us to propose them as effective and promising active layer luminescent materials for artificial lighting applications.

## 2. Results and Discussion

### 2.1. Synthesis and Characterization of Compounds

The synthesis of the ancillary ligand (N^N) was carried out starting from 1,10-phenanthrolin-5,6-dione as a precursor, as reported by Eisenberg and Paw [23], resulting a reddish solid in the case of the three ligands, after column purification, with a yield close to 50%. The obtention and purity of these compounds were corroborated by NMR spectroscopy. For further details, see the Supporting Information.

**Figure 1:**
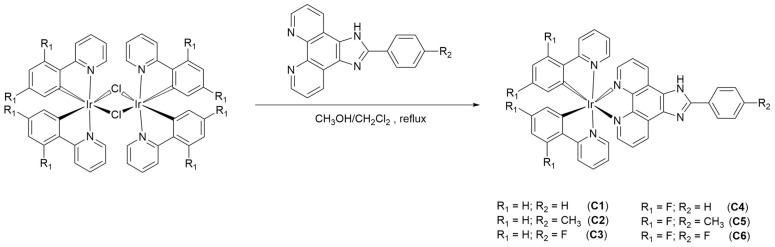
Synthesis of Ir(III) complexes with polypyridinic ligands and R_1_-phenylimidazo(4,5-*f*)1,10-phenanthroline.

Iridium (III) complexes **C1**–**C6** were synthesized following a general protocol (Figure 1), where IrCl_3_ x H_2_O reacts with the cyclometalated ligand, it forms the respective dimers corresponding to 2-phenylpyridine (ppy) in case of **C1**–**C3** complexes or 2-(2′,4′-difluorophenyl)pyridine (F_2_ppy) in case of **C4**–**C6**, by Nonoyama reaction [24]. Subsequently, this dimer reacted with the **L1**–**3** ligand to give the expected monomer, which later is precipitated as PF_6_-salt, favored with the addition of the respective equivalent of KPF_6_ and reaction with dimer.

**Figure 2:**
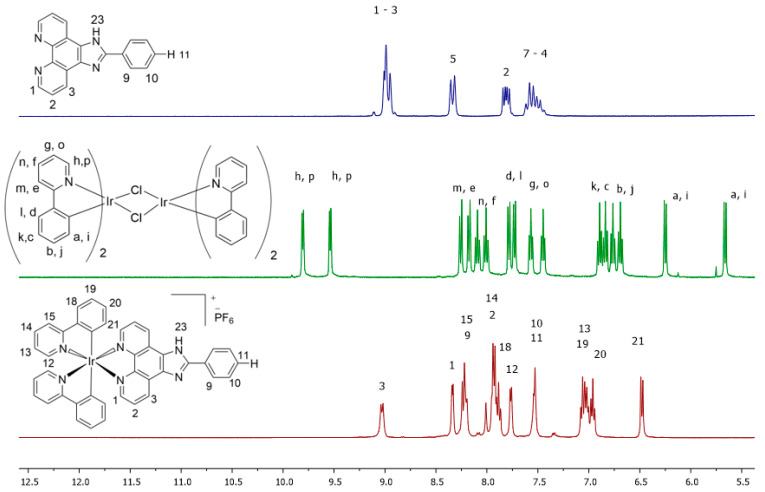
RMN of **L1** (**top**), dichloro-bridged iridium intermediate (**middle**) and complex **C1** (**bottom**).

The dimer intermediates obtained were confirmed by NMR, finding 16 and 12 signals in the aromatic zone that correspond to ppy and F_2_ppy ligands, respectively, which agree in terms of displacement and multiplicity as already reported [24]. Finally, complexes **C1**–**6** were structurally characterized by NMR, FT-IR, and HRMS evidencing the obtention of the compounds. The characterization confirms the monometallic structure shown in Figure 1, with two cyclometalated and one ancillary ligand. The complexes were obtained with a yield between 63 and 70%, like the analogous complex with the phenatroline derivate ligand. When comparing the ^1^H-NMR spectroscopy for **C1**, and its respective dimer and N^N ligand (Figure 2), a clear shift for hydrogens adjacent to the Ir-N bond in **L1**, labeled as 1, is observable, from 8.99 ppm to 8.35 ppm. This behavior verifies its inclusion in the final complex. Likewise, for ([Ir(ppy)_2_(μ-Cl)_2_], the lowest field signals, (labeled h/p) a clear up-shift is noticeable due to proximity to another Ir-N bond in the final complex (9.81/9.53 ppm to 7.75 ppm). A loss of the asymmetry present in the dimer implies cleavage of the bridging chlorine atoms, as only one of the sets of symmetrical Ir(ppy)_2_ signals is present in the **C1** complex. 

For all complexes, the characterization by infrared spectroscopy shows the characteristic bands of PF_6_ counterion around 843 and 557 cm^−1^ [25].

The characterization of all complexes within the same series via NMR spectroscopy does not exhibit significant differences among the various complexes, primarily due to the high symmetry inherent in this system, as depicted in Figure 2 for **C1**. However, in the aliphatic region, complexes **C2** and **C5** display singlets at 2.37 ppm and 2.43 ppm, respectively. This observed distinction aligns with the electron-withdrawing effect of 2-(2′,4′-difluorophenyl), as evidenced in analogous systems [26,27].

### 2.2. Electrochemical Behavior

The electrochemical behavior of the six complexes (**C1**–**6**) was determined by cyclic voltammetry in acetonitrile at room temperature using Ag/AgCl as the reference electrode and ferrocene as the standard for all measurements (see Supporting Information Figure S18). The values of the oxidation/reduction potentials (reported vs. Fc^+^/Fc) and the experimental HOMO-LUMO energy gaps, calculated as the difference between the oxidation and reduction potentials, are summarized in Table 1. The assignments of the redox processes were based on comparisons with electrochemical data previously reported for similar Ir(III) complexes [28,29]. While scanning towards positive potentials, **C1**–**C3** display a quasi-reversible process attributed to Ir(III)/(IV) oxidation, whereas the **C4**–**C6** complexes exhibit an irreversible behavior [3,25,30,31]. Quite expectedly, the oxidation potentials of the **C4**–**6** series (~1.1 V vs. Fc^+^/Fc) are more positive than those of the **C1**–**3** series (~0.9 V vs. Fc^+^/Fc) due the higher electron-accepting nature of the F_2_ppy ligands.

Under a cathodic scan, it is possible to observe two consecutive reduction processes that are attributable to the ancillary ligands [31]. As a matter of fact, these reductions occur in a very narrow range (~−1.8 and −2 V vs. Fc^+^/Fc, respectively), consistent with weak electronic effects exerted on the ancillary ligand by the R_2_ substituents of the cyclometalated ligand.

For all the oxidation and reduction processes, the effect of the R_1_ substituent is apparently negligible regardless of its electron-donating or -withdrawing character. This is evident as the reduction values remain similar within the **C1**–**C3** and **C4**–**C6** series and can be explained considering that the phenyl ring of the ancillary ligand is presumably twisted with respect to the phenanthroline moiety, so that the LUMO of the complexes is not appreciably located on the substituted phenyl ring [32,33]. Consistent with these considerations, similar redox gaps can be determined within the **C1**–**C3** and **C4**–**C6** subgroups (see Table 1) and the larger values for **C4**–**C6** series than **C1**–**C3** mainly reflect the energy of the HOMO in the fluorinated complexes due to the stabilization imparted by the R_2_ substituents.

### 2.3. Photophysical Properties

**Figure 3:**
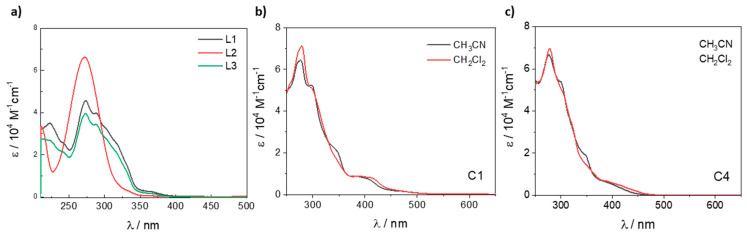
Absorption spectra of (**a**) **L1**–**3** in methanol (**b**) **C1** and (**c**) **C4** complexes in acetonitrile and dichloromethane.

Figure 3 shows the absorption spectra of ligands, **C1** and **C4** complexes as representative examples of each Ir(III) complex subgroup according to the different cyclometalating ligand (**C1**–**C3** using ppy and **C4**–**6** using F_2_ppy; see Supporting Information for the absorption spectra of the remaining complexes). Table 2 summarizes the absorption properties for the whole series of complexes **C1**–**6**. In the case of ligands, the absorption bands in the ultra-violet region between 250 and 340 nm are ascribed to intense spin-allowed (π→π*) transitions of 40,000–60,000 M^−1^ cm^−1^, being more intense for **L2**, with a low shoulder near to 350 nm, like observed in other imidazo-phenanthroline ligands [34].

For all complexes, intense absorption bands are observed in the UV region approximately between 250 and 320 nm (ε ~ 60,000–80,000 M^−1^ cm^−1^), assigned to spin-allowed π–π* transitions involving both the ancillary and the cyclometalating ligands [33]. In addition, all complexes showed absorption bands in the range 350–420 nm (ε ~ 5000–11,000 M^−1^ cm^−1^), attributable to a combination of ^1^MLCT (metal-to-ligand charge transfer) transitions from the iridium(III) center to the ancillary ligand and ^1^LLCT (ligand-to-ligand charge transfer) transitions from the C^N to the N^N ligand [35]. As can be seen in Table 2, in the case of the **C4**–**6** complexes a slightly blue shift of these latter bands is observed in comparison with the same patterns in **C1**–**3**, which can be related to the electron-withdrawing character of R_2_ in the cyclometalating ligand, which is in agreement with the electrochemical gaps experimentally calculated (see above, Table 1) [36]. Moreover, for all the complexes, it is also possible to observe weak bands above 450 nm (ε < 5000 M^−1^ cm^−1^) attributable to spin-forbidden ^3^MLCT transitions which are enabled, thanks to the high spin-orbit coupling of the iridium metal [37]. When comparing the lower energy absorptions for each complex in different solvents, a slight red-shift (*ca* 10 nm) is apparent when moving from acetonitrile to dichloromethane.

This solvatochromic effect is characteristic of charge transfer transitions (MLCT/LLCT) [37,38]. The luminescence properties of the **C1**–**6** complexes were then studied both in solution and in the solid state at 77 K. Figure 3 depicts the emission spectra of all complexes, while Table 3 presents the relevant photophysical data. It should be highlighted that for complexes **C1**–**6**, the emission profile is independent of the excitation wavelength.

**Figure 4:**
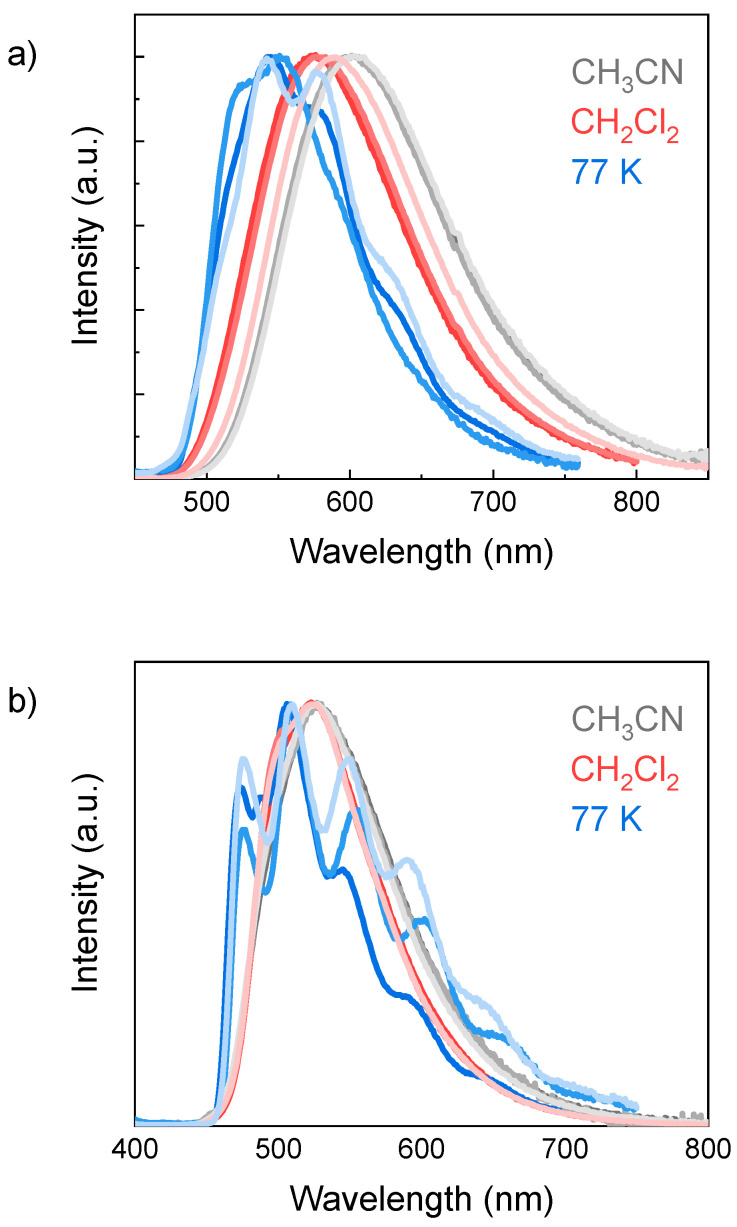
Emission spectra of (**a**) **C1**–**3** (from dark to light) and (**b**) **C4**–**6** (from dark to light) complexes in acetonitrile, dichloromethane, and 77 K glassy matrix.

Complexes **C1**–**3** (Figure 4a) present broad emission profiles in fluid solution at room temperature. The emission maximum falls at ~600 nm in acetonitrile, whereas it is blue-shifted (~580 nm) in dichloromethane. This solvatochromic effect is characteristic of excited states of charge transfer nature and, accordingly, the luminescence of complexes **C1**–**3** can be safely assigned to phosphorescence from triplet excited states of ^3^MLCT or ^3^LLCT nature or a mixture of both, as typically described for phenanthroline-derived cationic Ir(III) complexes [38]. Consistent with this attribution, the emission bands of complexes **C1**–**3** measured in the glassy matrix at 77 K are additionally blue-shifted with respect to solution conditions (maxima at ~540 nm) due to the peculiar rigidochromic effect of the ^3^MLCT/^3^LLCT-excited states [39].

In the case of complexes **C4**–**6**, emission is observed at higher energies than the parent complexes **C1**–**3**, which is consistent with the higher stabilization of the HOMO in the iridium(III) complexes involving the fluorinated cyclometalated ligands and the larger redox gap experimentally determined via electrochemical assays (Table 1) [40]. As a matter of fact, the luminescence in acetonitrile solution occurs at ~530 nm, while in dichloromethane it occurs at ~520 nm. The small solvatochromic effect experienced by the luminescence of complexes **C4**–**6** very likely suggests that for the latter, the emission may take place from an excited state admixture involving ^3^MLCT/^3^LLCT-excited states and an ^3^LC state on the ancillary ligand [41]. The observation of a structured emission pattern in the same wavelength range in the rigid matrix at 77 K (Figure 4b) further supports this hypothesis. As a matter of fact, the ^3^MLCT/^3^LLCT state is expected to increase in energy with respect to room temperature conditions, thus leading to pure ligand-based phosphorescence.

Quantum yields in the range 0.16–0.18 and 0.31–0.36 were measured for complexes **C1**–**3** in degassed acetonitrile and dichloromethane solutions, respectively. In the case of complexes **C4**–**6**, on the other hand, improved luminescence yields were recorded, with values in the range 0.27–0.39 in acetonitrile and 0.60–0.63 in dichloromethane. The emission enhancement with the decrease in solvent polarity can be qualitatively explained based on energy gap law arguments [42,43].

Time-resolved emission measurements were then performed on the whole set of complexes. For the **C1**–**3** series, both in fluid solution and at 77 K, the luminescence decays can be well fitted using a single exponential function (see Supporting Information and Table 3). Lifetimes between 530–630 ns and 770–960 ns can be extracted for acetonitrile and dichloromethane solutions, respectively. The increase in the lifetime from acetonitrile to dichloromethane parallels the increase in quantum yield and can be still associated with the deceleration of the radiationless transition upon decreasing solvent polarity (viz., energy-gap law). As a matter of fact, comparable radiative constants can be calculated for both solvent conditions (*k*_P_ ~ 3 × 10^5^ and ~4 × 10^5^ s^−1^ for acetonitrile and dichloromethane, respectively), while a decrease in the non-radiative constant can be estimated when moving from acetonitrile (average *k*_ISC_ ~ 1.4 × 10^6^ s^−1^ for **C1**–**3**) to dichloromethane (average *k*_ISC_ ~ 7.6 × 10^5^ s^−1^ for **C1**–**3**). In the glassy matrix at 77 K, the lifetimes substantially increase with values up to 4.47–5.48 μs. These values are characteristic of the excited states of ^3^MLCT/^3^LLCT nature, thus confirming the attribution previously made.

In the case of complexes **C4**–**6**, the luminescence decays at room temperature in fluid solution were also fitted using single exponential functions with lifetimes in the order of μs (see Supporting Information and Table 3). Similar values were obtained for both acetonitrile and dichloromethane solvents (in the range 4.66–5.67 μs and 3.95–5.02 μs, respectively). The radiative constants calculated for complexes **C4**–**6** (average *k*_P_ ~6.7 × 10^4^ and ~1.4 × 10^5^ s^−1^ for acetonitrile and dichloromethane, respectively) are appreciably lower than those previously estimated for **C1**–**3** (see above). This is consistent with an important ^3^LC character of the emitting excited state in complexes **C4**–**6**, thus confirming a mixed ^3^LC/^3^MLCT/^3^LLCT nature at room temperature, as previously envisioned. Interestingly, the luminescence decays measured for complexes **C4**–**6** in the rigid matrix at 77 K show two time-components in the μs time scale (Table 3). The observation of a long time constant in the order of 40–60 μs strongly points towards a dominant LC phosphorescence at low temperature, while the presence of two time components reflects a slow equilibration kinetics between the ^3^LC and ^3^MLCT/^3^LLCT-excited states under these conditions. 

For both steady-state and time-resolved luminescence measurements, no remarkable differences were appreciated upon changing the R_2_ group, still suggesting a minor effect of the differently substituted phenyl of the ancillary N^N ligand in the photophysics of the investigated iridium(III) complexes.

### 2.4. Electroluminescent Properties (EL)

Considering the interesting luminescence properties of complexes **C1**–**6**, LEC devices were fabricated following previously reported protocols [44], as described in the experimental section. The electroluminescence (EL) properties of each **C1**–**6** complex as an emissive layer in a ITO/PEDOT:PSS/Ir complex:EMIM-PF_6_(4:1)/Ga:In configuration are given in Table 4. Light emission was observed using all complexes with a rapid increase in luminance. A luminance maximum was reached after five minutes of device operation upon gradually increasing the voltage.

The devices exhibit low turn-on voltage, with the lowest value of 2.9 V being observed for **C2** and the highest value of 4.4 V for the **C4** complex. Complexes **C2** and **C5**, featuring methyl groups on the ancillary ligand, exhibit the lowest turn-on voltages of each subgroup. Recently, Cao et al. reported improved electroluminescence efficiencies for related iridium(III) complexes upon the insertion of methyl groups in the ancillary ligand and attributed this enhancement to the minimization of intermolecular interactions in the solid state [45].

**Figure 5:**
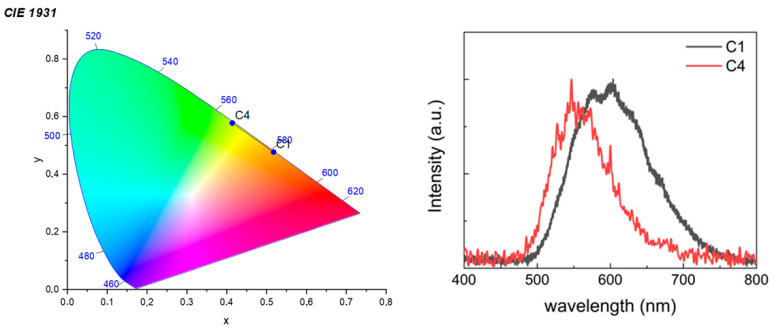
CIE graph (**left**) and electroluminescent spectra (**right**) of complexes **C1** and **C4** (at 15 and 8 V, respectively).

Figure 5 shows the CIE (International Commission on Illumination) graph and the electroluminescence spectra for **C1** and **C4** as examples of the two series of complexes. The **C1**–**3** series shows yellow-orange electroluminescent with CIE coordinates near (0.41, 0.57) with an electroluminescence maximum located between 596 and 579 nm, while electroluminescence from **C4**–**6** displays a green-yellow color, with CIE coordinates near (0.52, 0.47) for all series with maxima in the range 528–523 nm, akin to solution conditions (see above). Surprisingly, the luminance of **C1**–**3** is approximately 10 times higher than that measured for the **C4**–**6** series, showing an opposite trend with respect to the emission quantum yield in solution (see above). This can be associated with possible differences in the solid state structure of the complexes within the active layer due to the presence of the different cyclometalated ligands with flour atoms and to the long excited state lifetime for the **C4**–**6**, both potentially leading to a detrimental quenching phenomena. In addition, the **C1**–**3** series showcases a superior degree of reversibility based on voltage criteria, with ∆Ep values averaging a mere 51 mV, resulting in its capacity to act as an effective charge transport material, and thereby resist electrochemical degradation processes having an impact on device lifetimes and luminesce maxima [44].

While the devices exhibit relatively low turn-on voltages compared to other [Ir(R_2_-C^N)_2_(N^N)]^+^-type complexes [3,44], they have not surpassed the luminance of some more prominent counterparts in the literature that exceed 7000 cd m^−2^ [2]. As previously mentioned, this outcome is not solely or at least not directly attributed to properties predictable through their photophysical properties in solution. Rather, it is linked to a combination of effects within the device as we observed with this series of complexes.

## 3. Experimental Section

### 3.1. General Information and Materials

Commercially available reagents and solvents were used, unless otherwise specified. Ligands **L1**–**3** were synthesized according to previously reported methodologies [46]. Iridium dimers ([Ir(ppy)_2_(μ-Cl)_2_] and [Ir(F_2_ppy)_2_(μ-Cl)_2_]) were synthesized according to previous literature procedures [24,47], so were the complex **C1** [26]. One-dimensional and two-dimensional NMR measurements were performed in a Bruker spectrophotometer, model AV 400 MHz. Chemical shifts are presented in parts per million relative to TMS [^1^H and ^13^C, δ (SiMe_4_) = 0] or an external standard [δ (CFCl_3_) = 0 for ^19^F NMR]. HR-MS(ESI) experiments were carried out using a ThermoFisher Scientific Plus Orbitrap mass Spectrometer, with positive polarity and ionization voltage equal to 4 kV. The FT-IR spectra were recorded on a Shimadzu IRTracer 100 Fourier transform spectrophotometer, on KBr pellets in the range of 4000 to 500 cm^−1^. Cyclic voltammetry was measured on a CH Instruments model CHI-620C potentiostat using platinum as the working electrode, Ag/AgCl (ferrocene has been used as the standard for all measurements) as the reference electrode and a Pt wire as the counter electrode. Measurements were carried out with a 1 mM concentration of the complexes in CH_3_CN and 1 M of tetrabutylammonium hexafluorophosphate (TBAPF_6_) as the supporting electrolyte at a scan rate of 0.1 V s^−1^. The UV-Vis spectra were registered using a Shimadzu UV-Vis spectrometer, model 1900. To determine the molar extinction coefficient, a calibration curve was performed in CH_3_CN with concentrations ranging from 1 × 10^−5^ to 5 × 10^−5^ mol/L. Photoluminescence spectra were obtained on an Edinburgh Instrument spectrofluorometer. Solutions of the compounds were previously degassed with nitrogen for approximately 20 min. The emission quantum yields were calculated using a relative method according to a description in the literature [48]. Additionally, 77 K luminescence measurements were performed by freezing alcoholic solutions (ethanol/methanol, 4/1) of complexes and ligands.

**General synthetic procedure of complexes C1–6.** Two equivalents of the corresponding ligand (**L1**–**3**) and one equiv. of the respective bimetallic precursor [Ir(R_1_-ppy)_2_(μ-Cl)]_2_, with R_2_ = H or F, were dissolved in 50 mL of MeOH/CH_2_Cl_2_ (1:3). The mixture was stirred and refluxed for 12 h under a nitrogen atmosphere in darkness. Then, the volatiles were removed under reduced pressure, and 500 mL of water was added to the crude product. The mixture was filtered, and two equivalents of KPF_6_ were added to the obtained solution, precipitating a yellow-orange solid. This solid was filtered and washed with water, dried, and re-precipitated through CH_2_Cl_2_/diethyl ether [26,49].

**Complex C2.** Yellow-orange colored solid with a yield of 63%. ^1^H NMR (400 MHz, acetone d_6_, 298 K): δ ppm 9.03 (d, *J* = 7.9 Hz, 2H), 8.30 (d, *J* = 4.7 Hz, 2H), 8.23 (d, *J* = 8.2 Hz, 2H), 8.08 (d, *J* = 7.5 Hz, 2H), 7.93 (d, *J* = 7.7 Hz, 2H), 7.89 (t, *J* = 7.5 Hz, 4H), 7.76 (d, *J* = 5.6 Hz, 2H), 7.30 (d, *J* = 7.6 Hz, 2H), 7.06 (t, *J* = 7.8 Hz, 2H), 7.07–6.98 (m, 2H), 6.96 (t, *J* = 7.3 Hz, 2H), 6.48 (d, *J* = 7.5 Hz, 2H), 2.37 (s, 3H). ^13^C NMR (101 MHz, Acetone-*d*_6_, 298 K) δ 167.84, 153.21, 150.48, 149.46, 148.82, 144.69, 144.27, 140.62, 138.58, 132.12, 131.78, 130.37, 129.69, 126.54, 124.93, 123.53, 122.55, 119.85, 20.56. ^31^P NMR (162 MHz, Acetone-*d*_6_, 298 K) δ 144.20 (hept, *J^P-F^* = 708.0 Hz). ^19^F NMR (376 MHz, Acetone-*d*_6_, 298 K) δ −72.39 (d, *J^F-P^* = 708.0 Hz). HRMS (ESI): *m/z* [M]^+^ for C_42_H_30_IrN_6_: calc: 811.2161; found: 811.2202.

**Complex C3.** Yellow-orange colored solid with a yield of 66%. ^1^H NMR (400 MHz, acetone d_6_, 298 K): δ ppm 9.08 (dd, *J* = 8.3, 1.1 Hz, 2H), 8.34 (dd, *J* = 5.0, 1.3 Hz, 2H), 8.29 (dd, *J* = 8.8, 5.3 Hz, 2H), 8.24 (d, *J* = 8.1 Hz, 2H), 7.95 (dt, *J* = 11.4, 4.3 Hz, 4H), 7.92–7.87 (m, 2H), 7.75 (d, *J* = 5.6 Hz, 2H), 7.31 (t, *J* = 8.8 Hz, 2H), 7.07 (td, *J* = 7.7, 1.0 Hz, 2H), 7.04–6.99 (m, 2H), 6.96 (tt, *J* = 9.8, 4.9 Hz, 2H), 6.47 (d, *J* = 6.9 Hz, 2H). ^13^C NMR (101 MHz, Acetone-*d*_6_, 298 K) δ 167.83, 163.90 (d, *J^C-F^* = 249.1 Hz), 152.22, 150.43, 149.22 (d, *J^C-F^* = 51.7 Hz), 138.59, 132.13, 131.78, 130.37, 128.89 (d, *J^C-F^* = 8.8 Hz), 126.73, 124.93, 123.53, 122.56, 119.85, 116.05 (d, *J^C-F^* = 22.4 Hz). ^31^P NMR (162 MHz, Acetone-*d*_6_, 298 K) δ −144.22 (hept, *J^P-F^* = 708.0 Hz). ^19^F NMR (376 MHz, Acetone-*d*_6_, 298 K) δ −72.40 (d, *J^F-P^* = 708.0 Hz), −111.34. HRMS (ESI): *m/z* [M]^+^ for C_41_H_27_FIrN_6_: calc: 815.1910; found: 815.1954.

**Complex C4.** Yellow-colored solid with a yield of 66%. ^1^H NMR (400 MHz, acetone d_6_, 298 K): δ ppm 9.25 (d, *J* = 8.3 Hz, 2H), 8.51 (d, *J* = 4.9 Hz, 2H), 8.42 (d, *J* = 8.5 Hz, 2H), 8.31 (d, *J* = 7.0 Hz, 2H), 8.10 (dd, *J* = 8.2, 5.1 Hz, 2H), 8.02 (t, *J* = 7.9 Hz, 2H), 7.84 (d, *J* = 5.7 Hz, 2H), 7.67–7.53 (m, 3H), 7.11 (t, *J* = 6.6 Hz, 2H), 6.91–6.75 (m, 2H), 5.93 (dd, *J* = 8.5, 1.8 Hz, 2H). ^13^C NMR (101 MHz, Acetone-*d_6_*) δ 163.91 (d, *J^C-F^* = 7.0 Hz), 163.81 (dd, *J^C-F^* = 217.5, 12.7 Hz), 161.24 (dd, *J^C-F^* = 221.4, 12.6 Hz), 153.37, 150.02, 149.47, 144.55, 139.68, 132.82, 130.54, 129.56, 129.19, 128.14, 127.14, 126.66, 124.06, 113.91 (dd, *J^C-F^* = 17.7, 2.9 Hz), 98.80 (t, *J^C-F^* = 27.1 Hz). ^31^P NMR (162 MHz, Acetonitrile-*d*_3_, 298 K) δ −144.26 (hept, *J^P-F^* = 707.5 Hz). ^19^F NMR (376 MHz, Acetonitrile-*d*_3_, 298 K) δ −72.59 (d, *J^F-P^* = 707.6 Hz), −107.86 (d, *J^F-F^* = 10.6 Hz), −110.16 (d, *J^F-F^* = 10.5 Hz). HRMS (ESI): *m/z* [M]^+^ for C_41_H_24_F_4_IrN_6_: calc: 869,1628; found: 869,1688.

**Complex C5.** Yellow-colored solid with a yield of 65%. ^1^H NMR (400 MHz, Acetone-*d*_6_, 298 K) δ 13.38 (broad, 1H), 9.19 (broad, 2H), 8.49 (broad, 2H), 8.40 (d, *J* = 8.4 Hz, 2H), 8.17 (d, *J* = 7.8 Hz, 2H), 8.07 (broad, 2H), 8.00 (t, *J* = 8.0 Hz, 2H), 7.83 (d, *J* = 5.8 Hz, 2H), 7.41 (d, *J* = 7.8 Hz, 2H), 7.09 (broad, 2H), 6.79 (ddd, *J* = 12.1, 9.3, 2.4 Hz, 2H), 5.92 (dd, *J* = 8.6, 2.4 Hz, 2H), 2.43 (s, 3H). ^13^C NMR (101 MHz, Acetone-*d*_6_, 298 K) δ 164.77 (d, *J^C-F^* = 6.9 Hz), 164.48 (dd, *J^C-F^* = 256.2, 12.5 Hz), 162.30 (dd, *J^C-F^* = 260.2, 13.0 Hz), 155.49, 155.43, 154.38, 150.88, 150.24, 140.55, 133.63, 130.66, 129.01 (t, *J^C-F^* = 3.7 Hz), 127.95, 127.58, 124.96, 124.43 (d, *J^C-F^* = 19.8 Hz), 114.78 (dd, *J^C-F^* = 17.8, 3.0 Hz), 99.66 (t, *J^C-F^* = 27.1 Hz), 21.41. ^31^P NMR (162 MHz, Acetone-*d*_6_, 298 K) δ −146.44 (hept, *J^P-F^* = 708.3 Hz). ^19^F NMR (376 MHz, Acetone-*d*_6_, 298 K) δ −72.42 (d, *J^F-P^* = 708.3 Hz), −107.81 (d, *J^F-F^* = 10.3 Hz), −110.12 (d, *J^F-F^* = 10.2 Hz). HRMS (ESI): *m/z* [M]^+^ for C_42_H_26_F_4_IrN_6_: calc: 883,1784; found: 883,1832.

**Complex C6.** Yellow-colored solid with a yield of 70%. ^1^H NMR (400 MHz, Acetone-*d*_6_, 298 K) δ 9.16 (dd, *J* = 8.3, 1.4 Hz, 2H), 8.48 (dd, *J* = 5.0, 1.2 Hz, 2H), 8.42 (dt, *J* = 8.3, 1.6 Hz, 2H), 8.16 (dd, *J* = 6.8, 3.0 Hz, 2H), 8.05–7.97 (m, 4H, H2), 7.86 (dd, *J* = 5.8, 1.4 Hz, 2H), 7.65–7.40 (m, 3H), 7.13 (ddd, *J* = 7.4, 5.8, 1.4 Hz, 2H), 6.81 (ddd, *J* = 12.2, 9.4, 2.4 Hz, 2H), 5.92 (dd, *J* = 8.5, 2.4 Hz, 2H). ^13^C NMR (101 MHz, Acetone-*d*_6_, 298 K) δ 164.78 (d, *J^C-F^* = 7.1 Hz), 164.49 (dd, *J^C-F^* = 256.0, 12.6 Hz), 162.31 (d, *J^C-F^* = 260.1, 12.6 Hz), 155.49, 155.43, 154.01, 150.92, 150.32, 145.40, 140.58, 133.78, 131.38, 130.18, 130.02, 129.00 (d, *J^C-F^* = 4.5 Hz), 127.98, 127.55, 125.39, 124.97, 124.45 (d, *J^C-F^* = 19.9 Hz), 114.78 (dd, *J^C-F^* = 17.6, 2.9 Hz), 99.68 (t, *J^C-F^* = 27.0 Hz). ^31^P NMR (162 MHz, Acetone-*d*_6_, 298 K) δ −146.45 (hept, *J^P-F^* = 708.1 Hz). ^19^F NMR (376 MHz, Acetone-*d*_6_, 298 K) δ −72.52 (d, *J^F-P^* = 707.1 Hz), −107.83 (d, *J^F-F^* = 10.7 Hz), −110.12 (d, *J^F-F^* = 10.8 Hz), −150.74. HRMS (ESI): *m/z* [M]^+^ for C_41_H_23_F_5_IrN_6_: calc: 887,1534; found: 887,1574.

### 3.2. Device Preparation and Measurement

The device preparation was carried out according to previous reports [50]. Poly(3,4-ethylenedioxythiophene):polystyrenesulfonate (PEDOT:PSS) was purchased from Sigma-Aldrich. Indium tin oxide (ITO)-coated glass plates, purchased from ossila (14–16 Ω^−1^), were used as a transparent substrate and were extensively cleaned using sonification in a 2-propanol bath. After drying, the ITOs were placed in a Plasma cleaner for 20 min at room temperature. The electroluminescent devices were prepared as follows. A 100 nm layer of filtered PEDOT:PSS was deposited at 1100 rpm for 60 s, the film thickness was determined using an optical profilometer Profilm 3D from Filmetrics. Then, a thin film of **C1**–**6** containing ionic liquid (1-ethyl-3-methylimidazolium hexafluorophosphate) (EMIM-PF_6_) in a 4:1 proportion was obtained in spin-coating equipment from acetonitrile solutions using concentrations of 20 mg mL^−1^ at 1100 rpm for 60 s, resulting in an 80 nm thick film. After spinning the inorganic layers, the samples were dried on a hot plate at 75 °C for 1 h. Ga:In eutectic cathode was deposited on the EMIM/iTMC layer in 1 cm^2^. A spectrophotometer model CCS200 was used to record the electroluminescence spectra of the devices and to obtain the respective CIE coordinates. Finally, a PCE_CR, 40 luxmeter was used to determine photometric magnitudes.

## 4. Conclusions

A series of six iridium(III) complexes have been prepared and characterized. Complexes **C1**–**3** with two ppy cyclometalated ligands and three different phenylimidazo(4,5-*f*)1,10-phenanthroline ancillary ligand display luminescence in solution of ^3^MLCT/^3^LLCT nature. On the other hand, the related complexes **C4**–**6**, featuring F_2_-ppy cyclometalated ligands in place of ppy, present intense and blue-shifted luminescence arising from an excited state admixture involving ^3^MLCT/^3^LLCT and ^3^LC-excited states. The complexes behave as effective active layers in LEC devices. At maximum voltage, the **C1**–**C3** complexes emit orange light, while the **C4**–**C6** complexes emit yellow-green light, with a turn-on close to 5 s. The **C1**–**C3** group had long on times with luminance near 25 Lx, while the second group exhibit lower luminance close to 4.0 Lx but high stability. On the other hand, the inclusion of methyl group in the ancillary ligand reduces the intermolecular interactions, which results in a lower turn-on voltage in devices. The modification of the ancillary ligands in the presented complexes does not imply significant changes in the electrochemical or photophysical properties, which, on the other hand, are mostly influenced by modifications of the cyclometalating ligands. These results contribute to the understanding of how structural alterations on iridium(III) complexes impact the performance in LEC devices.

## Figures and Tables

**Table 1:** Electrochemical properties of iridium (III) complexes (potentials vs. Fc^+^/Fc).

Complex	E_ox_ (V)	E_red,1_ (V)	E_red,2_ (V)	∆E (V)
**C1**	0.88	−1.81	−2.04	2.69
**C2**	0.89	−1.81	−2.06	2.70
**C3**	0.88	−1.80	−2.06	2.68
**C4**	1.10	−1.74	−1.98	2.84
**C5**	1.11	−1.75	−1.98	2.86
**C6**	1.12	−1.74	−1.98	2.86

**Table 2:** Summary of the absorption data.

	λ_abs_/nm	
Complex	CH_3_CN	CH_2_Cl_2_
**C1**	276, 297, 345, 384, 403	278, 298, 346 (sh), 388, 412
**C2**	279, 294 (sh), 381, 403	280, 299 (sh), 389, 414
**C3**	274, 295, 341 (sh), 381, 406	276, 296, 345 (sh), 387, 412
**C4**	277, 300, 347, 378	278, 303 (sh), 352, 387
**C5**	279, 301, 349, 384	281, 304 (sh), 358, 390
**C6**	277, 301 (sh), 346, 379	278, 303 (sh), 355, 390

**Table 3:** Summary of the luminescence data.

	λ_max_ (nm)			Φ *^b^*		τ/μs *^c^*		
Complex	CH_3_CN	CH_2_Cl_2_	77 K *^a^*	CH_3_CN	CH_2_Cl_2_	CH_3_CN	CH_2_Cl_2_	77 K *^a^*
**C1**	602	574	577,540	0.18	0.35	0.63	0.89	4.47
**C2**	600	576	550,524	0.16	0.36	0.58	0.96	5.48
**C3**	603	588	577,540	0.18	0.31	0.53	0.77	4.70
**C4**	528	522	544,507,473	0.39	0.60	4.66	3.95	7.5; 37.9
**C5**	528	525	553,509,474	0.27	0.61	5.67	5.02	13.6; 62.4
**C6**	527	523	549,508,475	0.35	0.63	4.99	4.57	12.5; 57.1

*^a^* 1/4 methanol/ethanol glassy matrix; *^b^* estimated using Ru(bpy)_3_^2+^ in water (Φ = 0.028) as standard for **C1**–**3** and fluorescein in 0.1 M NaOH (Φ = 0.96) as standard for **C4**–**6**; *^c^* excitation at 355 nm.

**Table 4:** Electroluminescent data of **C1**–**6**-based LEC devices.

iTMC	λ_EL_	V_turn.on_ (V) ^a^	L_max_ (Lx) ^b^	V_max_ (V)
**C1**	596	3.5	15.5	15
**C2**	587	2.9	21.7	14.7
**C3**	579	3.9	16.1	14
**C4**	527	4.4	3.5	8
**C5**	523	3	4.4	8.3
**C6**	528	3.5	4.1	7.9

^a^: Defined as the bias at brightness of 1 cd m^−2^. ^b^: associated with the maximum voltage.

## Data Availability

Data are contained within the article and Supplementary Materials.

## Supplementary Materials

The following supporting information can be downloaded at: https://www.mdpi.com/article/10.3390/molecules29010053/s1, you can find the Figure S1–S28. Refs [26,47,51,52,53,54] are cited in Supplementary Materials.
