# Supplementary material for: Synthesis and Characterization of Iridium(III) Complexes with Substituted Phenylimidazo(4,5-f)1,10-phenanthroline Ancillary Ligands and Their Application in LEC Devices
Source: Molecules. 2023 Dec 21;29(1):53. doi: 10.3390/molecules29010053 (PMC10779995; doi:10.3390/molecules29010053)
Supplement: Supplementary file 1 [file molecules-29-00053-s001.zip › molecules-2731922-supplementary.pdf]

Supporting Information

Experimental Section

All reagents and solvents commercially available were used as received, unless otherwise specified. Iridium dimer ($[Ir(R_2\text{-}ppy)_2(\mu Cl)]_2$), phenanthroline derivatives ligands, and **C1** complex were prepared as described in literature. [26,47,51–54]

Synthesis and Characterization of Compounds.

General synthesis of ligands L1-3.

A mixture of 40 mL of sulfuric acid and 20 mL of nitric acid were added to 1 equiv. (450.0 mg, 2.5 mmol) of 1,10- phenanthroline and 1.5 equiv. of KBr (445 mg, 3.75 mmol). The solution was left to reflux for 3 hours, poured over ice and neutralized with sodium hydroxide. After dilution with brine, the crude was extracted with CH_3Cl and dried with anhydrous sodium sulfate. Afterward, the solvent was reduced to precipitate the 1,10-phenantroline-5,6-dione using diethyl ether. After that, 1 equiv. (525.5 mg, 2.5 mmol) of 1,10-phenantroline-5,6-dione was mixed with 1 equiv. (300.4 mg, 2.5 mmol) of benzaldehyde, 2 equiv. (385.0 mg, 5 mmol) of ammonium acetate and 100 mL of acetic acid and taken to reflux for 2 hours. The resulting mixture was diluted in 400 mL of ice and water and neutralized using ammonium hydroxide. The precipitate was purified and extracted by chromatographic column using ethanol as eluent to afford 2-phenyl-1H-imidazo-(4,5-f)-1,10-phenanthroline (**L1**).

This procedure was repeated replacing benzaldehyde by 4-methylbenzaldehyde to obtain (4-methylphenyl)imidazo-(4,5-f)-1,10-phenanthroline (**L2**) and 4-flourobenzaldehyde to obtain 2-(4-flourofenyl)imidazo-(4,5-f)-1,10-phenanthroline (**L3**).

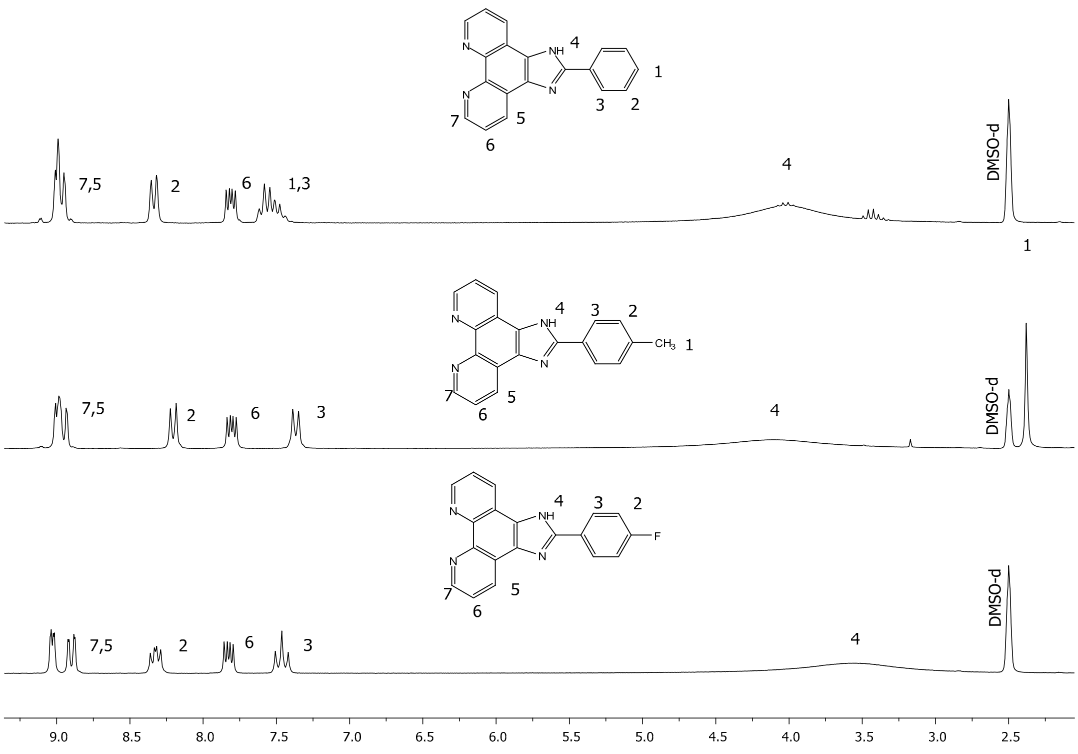

Figure S1. ^1H NMR (400 MHz, DMSO-d_6, 298 K) of L1-3

Synthesis of dichloro-bridged iridium intermediate [Ir(R_{1}-ppy)$_2$(μ-Cl)$_2$])

The precursor chloro-bridged iridium dimer complexes have been synthesized according to the following synthetic route.[6, 7] One equivalent (1000 mg, 3.349 mmol) of iridium(III) chloride hydrate was added to a solution of 2.2 eqiv. 2-phenylpyridine (1140 mg, 7.345 mmol) or 2-(2,4-difluorophenyl)pyridine (1404 mg, 7.345 mmol) in 100 mL of 2-methoxyethanol and 30 mL of water, and refluxed with stirring at 120°C under N_2 for 12 h, forming a green solid after cooling. This solid was collected via filtration, washed with water, dried, and re-precipitated from a chloroform:diethyl ether solution to yield the pure product of [Ir(ppy)$_2$(μ-Cl)$_2$]) and [Ir(F_2ppy)$_2$(μ-Cl)$_2$]), see Figure S2

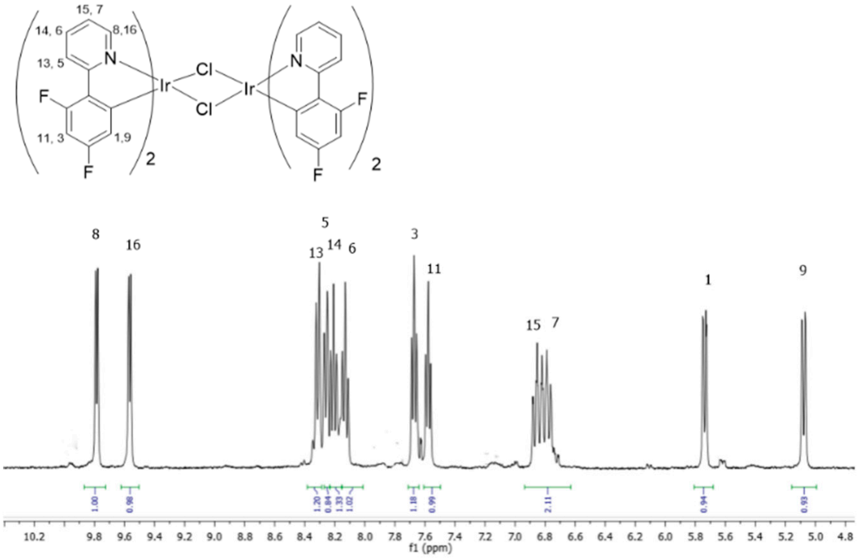

Figure S2. ^{1}H NMR (400 MHz, Acetone-d_6, 298 K) of intermediate of [Ir(F_2ppy)$_2$(μ-Cl)$_2$])

[6] M. S. Lowry, J. S. Goldsmith, J. D. Slinker, R. A. Pascal Jr., G. Malliaras, and S. Bernhard. *Chemistry of Materials* **2023** *35* (3), 1466-1466

[7] Nonoyama M. Benzo[h]quinolin-10-yl- N Iridium(III) Complexes. *Bull Chem Soc Jpn* **1974** 47 (3) : 767–8

General synthesis of Ir(III) complexes.

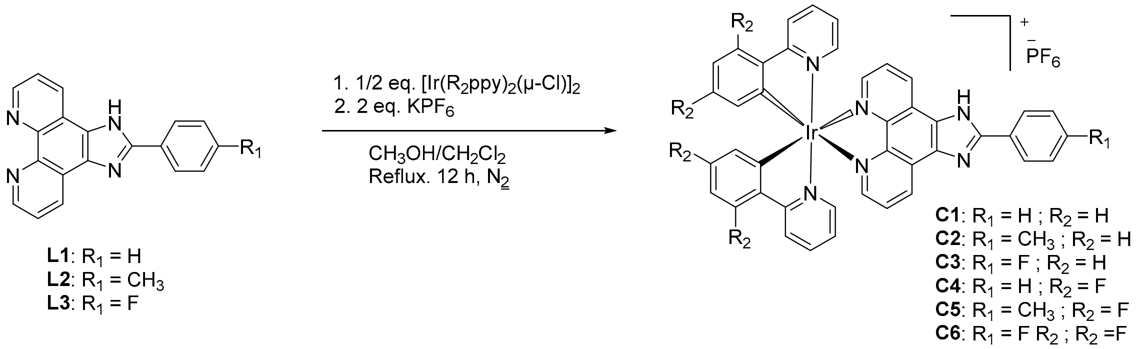

[(2-(4-methylphenyl)-1*H*-imidazo[4,5-*f*][1,10]phenanthroline)bis(2-phenylpyridine)Ir(III)]PF_6 (**C2**)

Two equiv. (37.2 mg, 0.12 mmol) of the *N,N* ligand (**L2,**) and one equiv. (73.0 mg, 0.06 mmol) of the respective bimetallic precursor $[Ir(ppy)_2(\mu\text{-}Cl)]_2$, were dissolved in 50 mL of $MeOH/CH_2Cl_2$ (1:3). The mixture was stirred and refluxed for 12 h under a nitrogen atmosphere in darkness. Then, the volatiles were removed under reduced pressure, and 500 mL of water was added to the crude product. The mixture was filtered, and two equivalents (22.1 mg, 0.12 mmol) of KPF_6 were added to the obtained solution, precipitating a yellow-orange solid. This solid was filtered and washed with water, dried, and re-precipitated through CH_2Cl_2/diethyl ether diffusion.

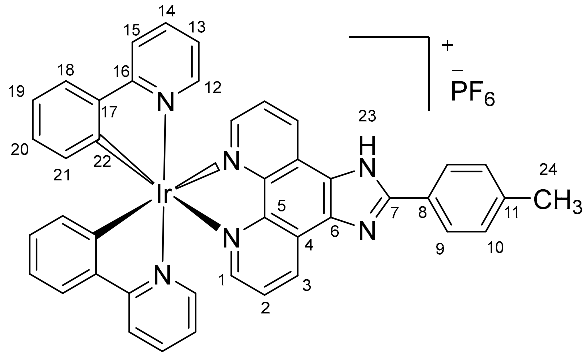

Yellow solid, 63 % yield.

^{1}H NMR (400 MHz, Acetone-d_6, 298 K) δ 9.03 (d, *J* = 7.9 Hz, 2H, H1), 8.30 (d, *J* = 4.7 Hz, 2H, H3), 8.23 (d, *J* = 8.2 Hz, 2H, H12), 8.08 (d, *J* = 7.5 Hz, 2H, H9), 7.93 (d, *J* = 7.7 Hz, 2H, H18), 7.89 (t, *J* = 7.5 Hz, 4H, H13, H2), 7.76 (d, *J* = 5.6 Hz, 2H, H15), 7.30 (d, *J* = 7.6 Hz, 2H, H10), 7.06 (t, *J* = 7.8 Hz, 2H, H19), 7.07 – 6.98 (m, 2H, H14), 6.96 (t, *J* = 7.3 Hz, 2H, H20), 6.48 (d, *J* = 7.5 Hz, 2H, H21), 2.37 (s, 3H, H24).

^{13}C NMR (101 MHz, Acetone-d_6, 298 K) δ 167.84 (C16), 153.21 (C7), 150.48 (C22), 149.46 (C20), 148.82 (C3), 144.69 (C5), 144.27 (C16), 140.62 (C11), 138.58 (C13), 132.12 (C1),

131.78 (C21), 130.37 (C20), 129.69 (C10), 126.54 (C9), 124.93 (C15), 123.53 (C14), 122.55 (C19), 119.85 (C12), 20.56 (C24).

^{31}P NMR (162 MHz, Acetone-d_6, 298 K) δ -144.20 (hept, $J^{P\text{-}F}$ = 708.0 Hz, PF_6).

^{19}F NMR (376 MHz, Acetone-d_6, 298 K) δ -72.39 (d, $J^{F\text{-}P}$ = 708.0 Hz, PF_6)

IR (KBr): ν/cm^{-1} = 3475 (N-H stretching), 1481 (C= stretching), 1361 (C-C stretching), 844 (P-F bending).

HRMS (ESI): m/z $[M]^+$ for $C_{42}H_{30}IrN_6$: calc: 811.2161; found: 811.2202.

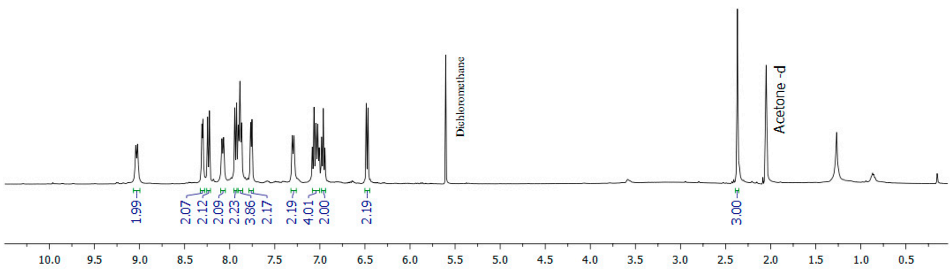

Figure S3. ^{1}H NMR (400 MHz, Acetone-d_6, 298 K)

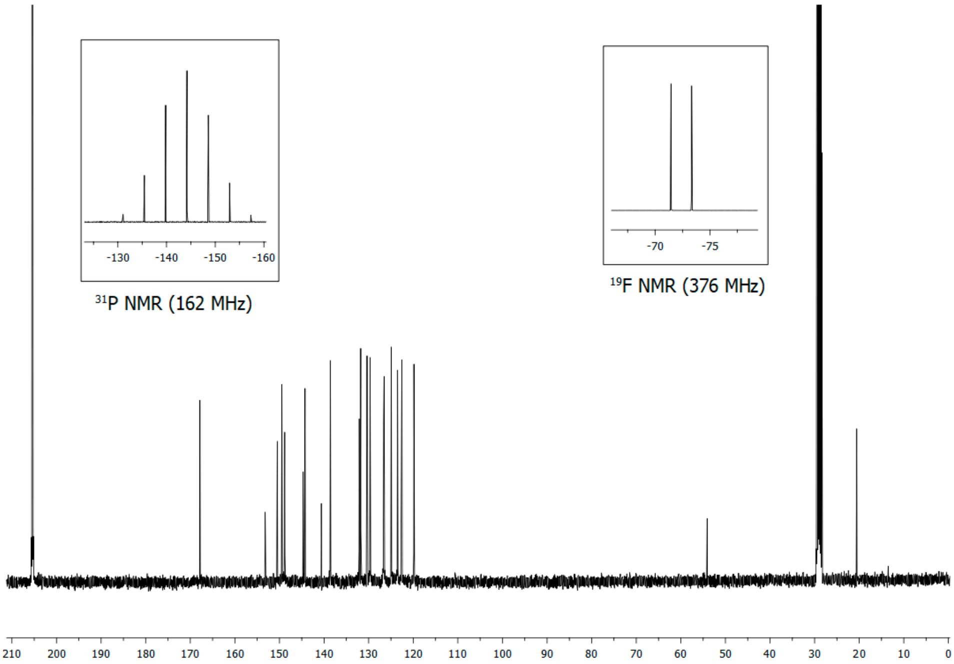

Figure S4. ^{13}C NMR (101 MHz, Acetone-d_6, 298 K) and inserts of ^{19}F NMR and ^{31}P NMR.

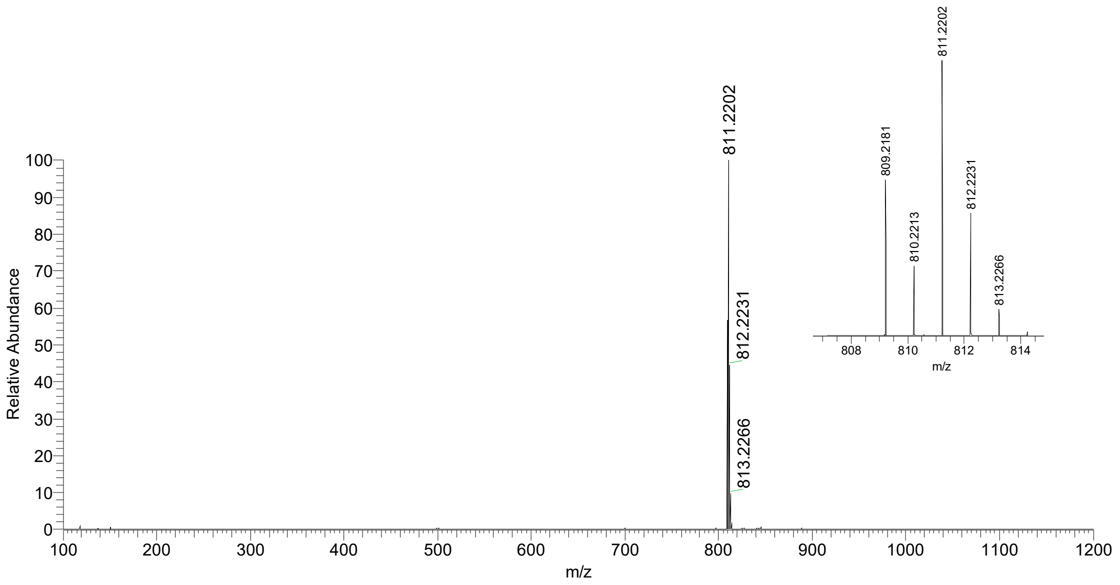

Figure S5. HRMS for **C2** (ESI – Positive Mode)

[(2-(4-fluorophenyl)-1*H*-imidazo[4,5-*f*][1,10]phenanthroline)bis(2-phenylpyridine)Ir(III)]PF_6 (**C3**). This complex was obtained using the same method as above, but using **L3**

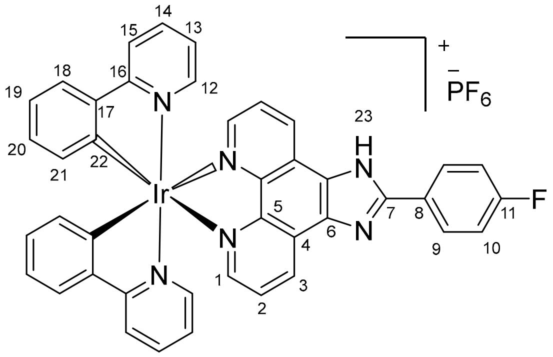

Yellow solid, 66 % yield.

^{1}H NMR (400 MHz, Acetone-*d*$_6$, 298 K) δ 9.08 (dd, *J* = 8.3, 1.1 Hz, 2H, H1), 8.34 (dd, *J* = 5.0, 1.3 Hz, 2H, H3), 8.29 (dd, *J* = 8.8, 5.3 Hz, 2H, H12), 8.24 (d, *J* = 8.1 Hz, 2H, H9), 7.95 (dt, *J* = 11.4, 4.3 Hz, 4H, H18, H2), 7.92 – 7.87 (m, 2H, H14), 7.75 (d, *J* = 5.6 Hz, 2H, H15), 7.31 (t, *J* = 8.8 Hz, 2H, H10), 7.07 (td, *J* = 7.7, 1.0 Hz, 2H, H19), 7.04 – 6.99 (m, 2H, H14), 6.96 (tt, *J* = 9.8, 4.9 Hz, 2H, H20), 6.47 (d, *J* = 6.9 Hz, 2H, H21).

^{13}C NMR (101 MHz, Acetone-*d*$_6$, 298 K) δ 167.83 (C16), 163.90 (d, $J^{C\text{-}F}$ = 249.1 Hz, C11), 152.22 (C7), 150.43 (C22), 149.22 (d, $J^{C\text{-}F}$ = 51.7 Hz, C20), 138.59 (C13), 132.13 (C1), 131.78 (C21), 130.37 (C20), 128.89 (d, $J^{C\text{-}F}$ = 8.8 Hz, C15), 126.73 (C9), 124.93 (C2), 123.53 (C19), 122.56 (C19), 119.85 (C12), 116.05 (d, $J^{C\text{-}F}$ = 22.4 Hz, C10).

^{31}P NMR (162 MHz, Acetone-*d*$_6$, 298 K) δ -144.22 (hept, $J^{P\text{-}F}$ = 708.0 Hz, PF_6).

^{19}F NMR (376 MHz, Acetone-d_6, 298 K) δ -72.40 (d, $J^{F\text{-}P}$ = 708.0 Hz, PF_6), -111.34 (s, F-Ph).

IR (KBr): ν/cm^{-1} = 3367 (N-H stretching), 1481 (C= stretching), 1226 (C-C stretching), 852 (P-F bending).

HRMS (ESI): m/z $[M]^+$ for $C_{41}H_{27}FIrN_6$: calc: 815.1910; found: 815.1954.

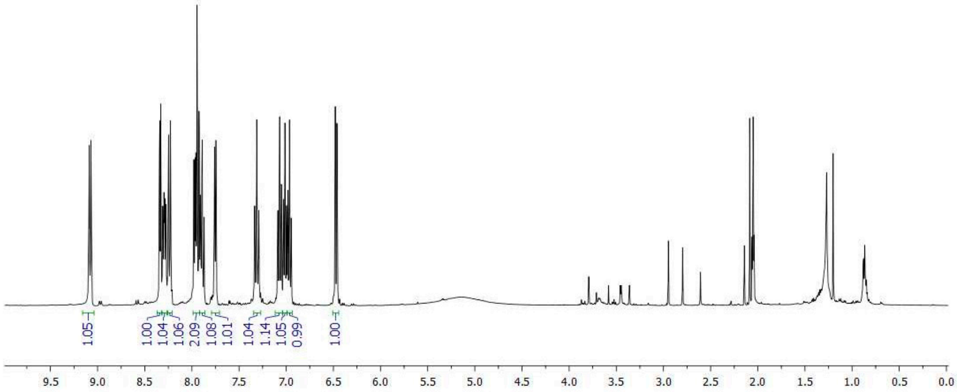

Figure S6. ^{1}H NMR (400 MHz, Acetone-d_6, 298 K)

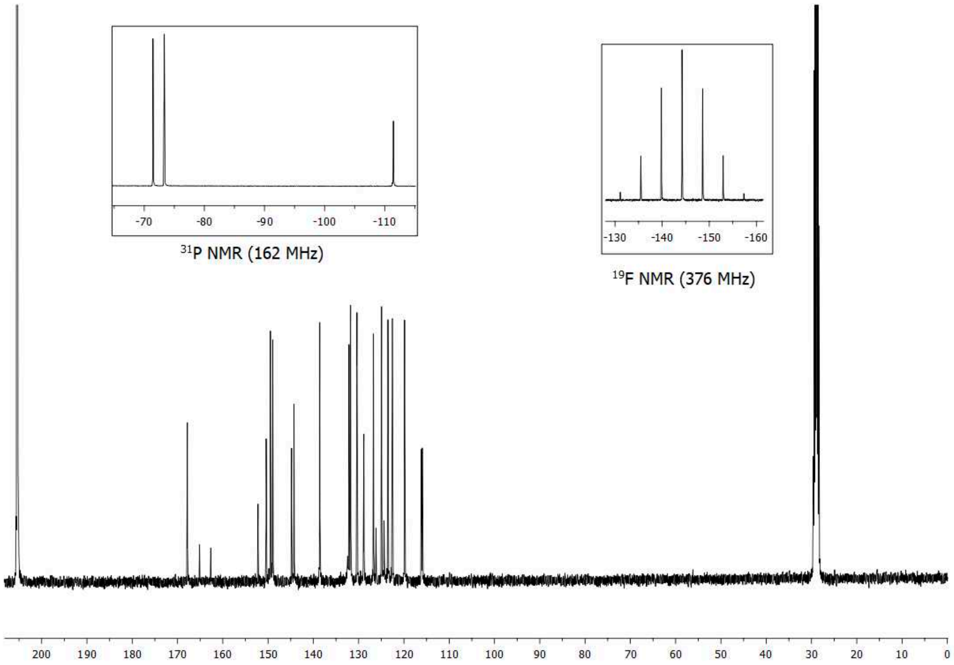

Figure S7. ^{13}C NMR (101 MHz, Acetone-d_6, 298 K) and inserts of ^{19}F NMR and ^{31}P NMR.

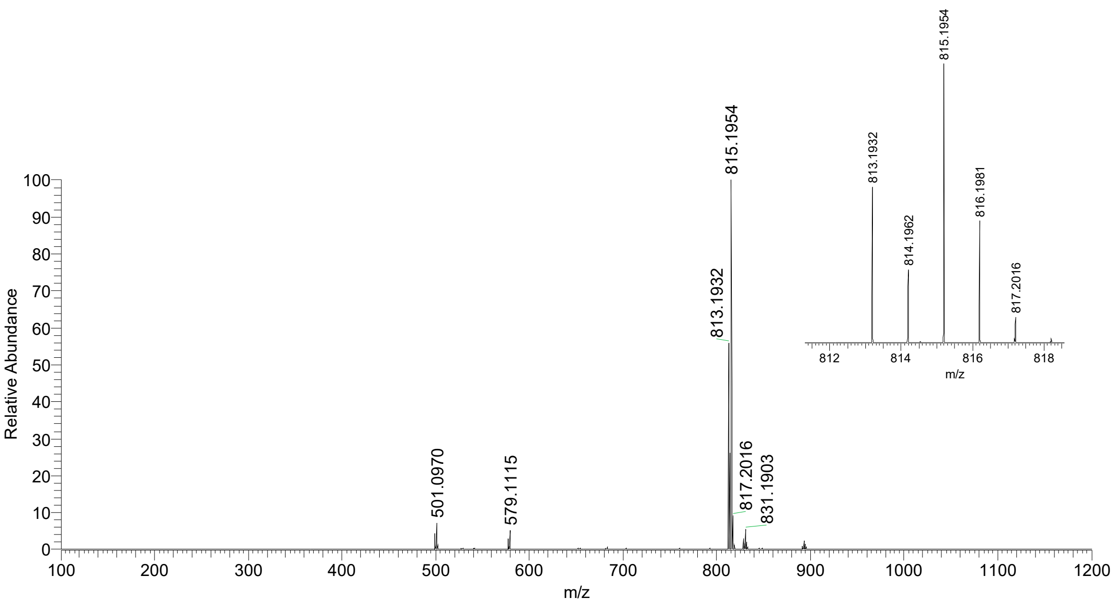

Figure S8. HRMS for **C3** (ESI – Positive Mode)

[(2-phenyl-1*H*-imidazo[4,5-*f*][1,10]phenanthroline)bis(2-(2,4-difluorophenyl)-pyridine)Ir(III)]PF_6 (**C4**) This complex was obtained using the same method as above, but using [Ir(F_2ppy)$_2$(μ-Cl)$_2$]) and **L1**

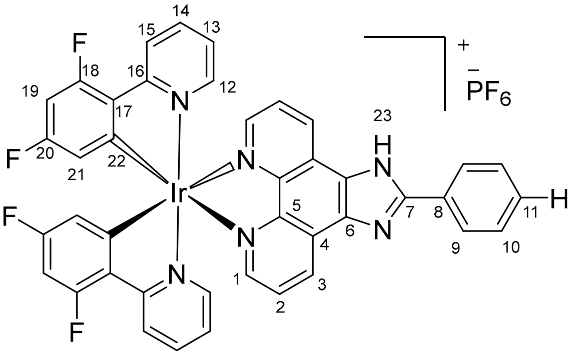

Yellow solid, 66 % yield.

^{1}H NMR (400 MHz, Acetone-d_6) , δ 9.25 (d, J = 8.3 Hz, 2H, H1), 8.51 (d, J = 4.9 Hz, 2H, H3), 8.42 (d, J = 8.5 Hz, 2H, H12), 8.31 (d, J = 7.0 Hz, 2H, H9), 8.10 (dd, J = 8.2, 5.1 Hz, 2H, H2), 8.02 (t, J = 7.9 Hz, 2H, H13), 7.84 (d, J = 5.7 Hz, 2H, H15), 7.67 – 7.53 (m, 3H, H10,11), 7.11 (t, J = 6.6 Hz, 2H, H14), 6.91 – 6.75 (m, 2H, H19), 5.93 (dd, $J^{H\text{-}F}$ = 8.5, 1.8 Hz, 2H, H21).

^{13}C NMR (101 MHz, Acetone-d_6) δ 163.91 (d, $J^{C\text{-}F}$ = 7.0 Hz, C16), 163.81 (dd, $J^{C\text{-}F}$ = 217.5, 12.7 Hz, C20), 161.24 (dd, $J^{C\text{-}F}$ = 221.4, 12.6 Hz, C18), 153.37 (C7), 150.02 (C15), 149.47 (C3), 144.55 (C5), 139.68 (C13), 132.82 (C1), 130.54 (C11), 129.56 (C11), 129.19 (C10), 128.14 (C17), 127.14 (C2), 126.66 (C9), 124.06 (C14), 113.91 (dd, $J^{C\text{-}F}$ = 17.7, 2.9 Hz, C21), 98.80 (t, $J^{C\text{-}F}$ = 27.1 Hz, C19).

^{31}P NMR (162 MHz, Acetonitrile-d_3, 298 K) δ - 144.26 (hept, $J^{P\text{-}F}$ = 707.5 Hz, PF_6).

^{19}F NMR (376 MHz, Acetonitrile-d_3, 298 K) δ -72.59 (d, $J^{F\text{-}P}$ = 707.6 Hz, PF_6), -107.86 (d, $J^{F\text{-}F}$ = 10.6 Hz, *p*-F-ppy), -110.16 (d, $J^{F\text{-}F}$ = 10.5 Hz, *o*-F-ppy).

IR (KBr): ν/cm^{-1} = 3421 (N-H stretching), 1516 (C=C stretching), 1404 (C-C stretching), 864 (P-F bending).

HRMS (ESI): m/z $[M]^+$ for $C_{41}H_{24}F_4IrN_6$: calc: 869,1628; found: 869,1688.

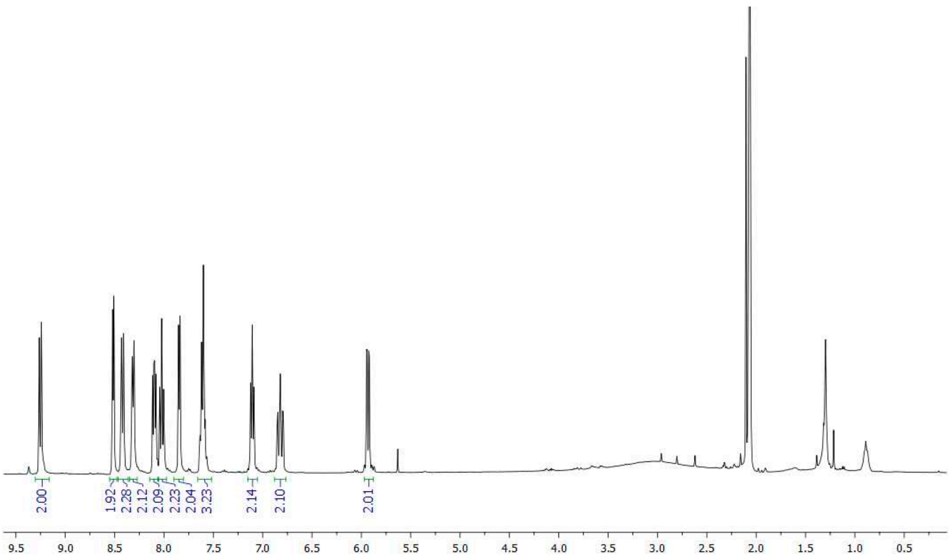

Figure S9. ^1H NMR (400 MHz, Acetone-d_6, 298 K)

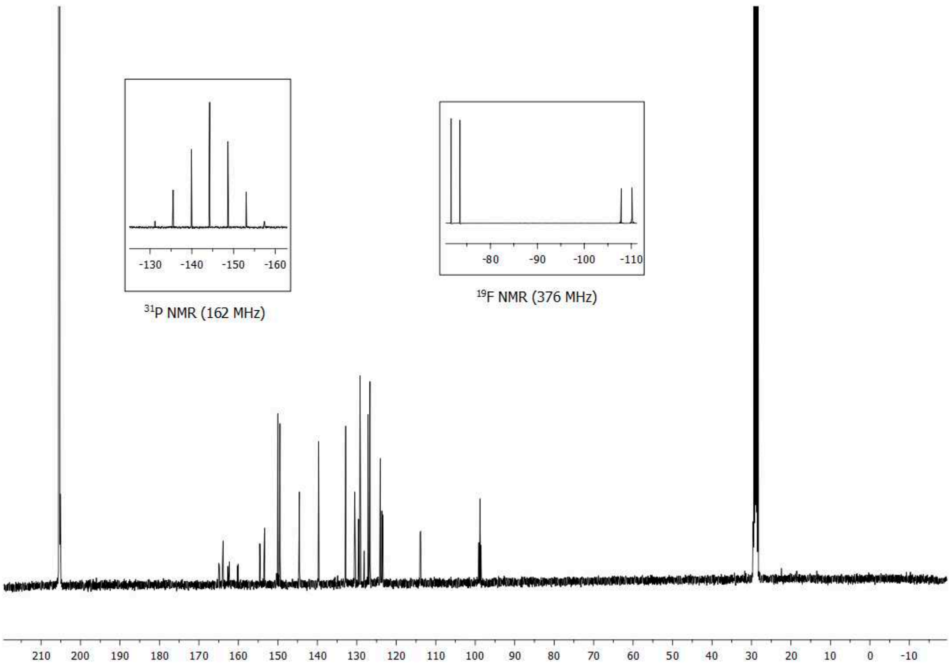

Figure S10. ^{13}C NMR (101 MHz, Acetone-d_6, 298 K) and inserts of ^{19}F NMR and ^{31}P NMR.

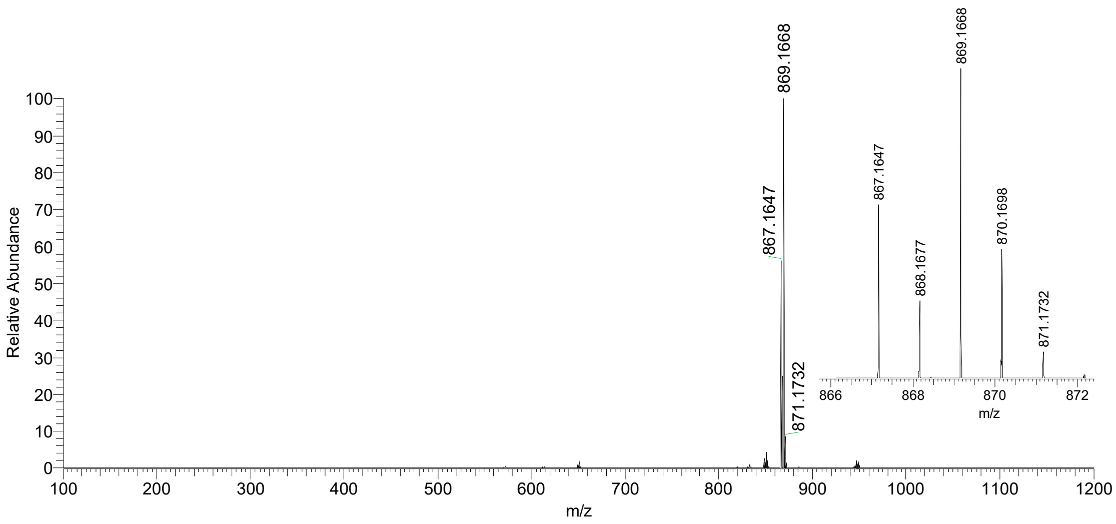

Figure S11. HRMS for **C4** (ESI – Positive Mode)

[(2-(*p*-tolyl)-1*H*-imidazo[4,5-*f*][1,10]phenanthroline)bis(2-(2,4-difluorophenyl)pyridine)Ir(III)]PF_6 (**C5**) This complex was obtained using the same method as above, but using [$Ir(F_2ppy)_2(\mu\text{-}Cl)_2$]) and **L2**.

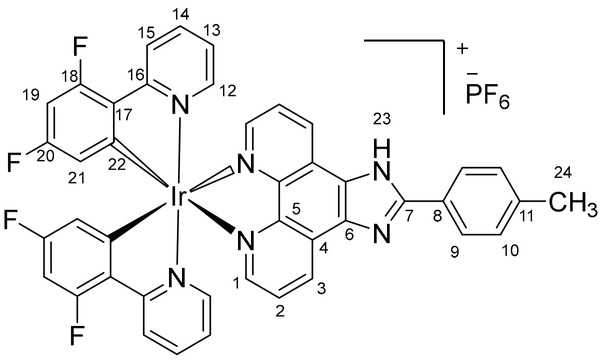

Yellow solid, 65 % yield.

^{1}H NMR (400 MHz, Acetone-d_6, 298 K) δ 13.38 (broad, 1H, H23), 9.19 (broad, 2H, H1), 8.49 (broad, 2H, H3), 8.40 (d, *J* = 8.4 Hz, 2H, H12), 8.17 (d, *J* = 7.8 Hz, 2H, H9), 8.07 (broad, 2H, H2), 8.00 (t, *J* = 8.0 Hz, 2H, H13), 7.83 (d, *J* = 5.8 Hz, 2H, H15), 7.41 (d, *J* = 7.8 Hz, 2H, H10), 7.09 (broad, 2H, H14), 6.79 (ddd, *J* = 12.1, 9.3, 2.4 Hz, 2H, H19), 5.92 (dd, *J* = 8.6, 2.4 Hz, 2H, H21), 2.43 (s, 3H, H24).

^{13}C NMR (101 MHz, Acetone-d_6, 298 K) δ 164.77 (d, $J^{C\text{-}F}$ = 6.9 Hz, C16), 164.48 (dd, $J^{C\text{-}F}$ = 256.2, 12.5 Hz, C20), 162.30 (dd, $J^{C\text{-}F}$ = 260.2, 13.0 Hz, C18), 155.49 (C6), 155.43 (C6b),

154.38 (C7), 150.88 (C15), 150.24 (C3), 140.55 (C13), 133.63 (C1), 130.66 (C10), 127.95 (C2), 127.58 (C9), 124.96 (C14), 124.43 (d, $J^{C\text{-}F}$ = 19.8 Hz, C12), 114.78 (dd, $J^{C\text{-}F}$ = 17.8, 3.0 Hz, C21), 99.66 (t, $J^{C\text{-}F}$ = 27.1 Hz, C19), 21.41 (C24).

^{31}P NMR (162 MHz, Acetone-d_6, 298 K) δ -146.44 (hept, $J^{P\text{-}F}$ = 708.3 Hz, PF_6).

^{19}F NMR (376 MHz, Acetone-d_6, 298 K) δ -72.42 (d, $J^{F\text{-}P}$ = 708.3 Hz, PF_6), -107.81 (d, $J^{F\text{-}F}$ = 10.3 Hz, *p*-F-ppy), -110.12 (d, $J^{F\text{-}F}$ = 10.2 Hz, *o*-F-ppy).

IR (KBr): ν/cm^{-1} = 3367 (N-H stretching), 1523 (C=C stretching), 1365 (C-C stretching), 860 (P-F bending).

HRMS (ESI): m/z $[M]^+$ for $C_{42}H_{26}F_4IrN_6$: calc: 883,1784; found: 883,1832.

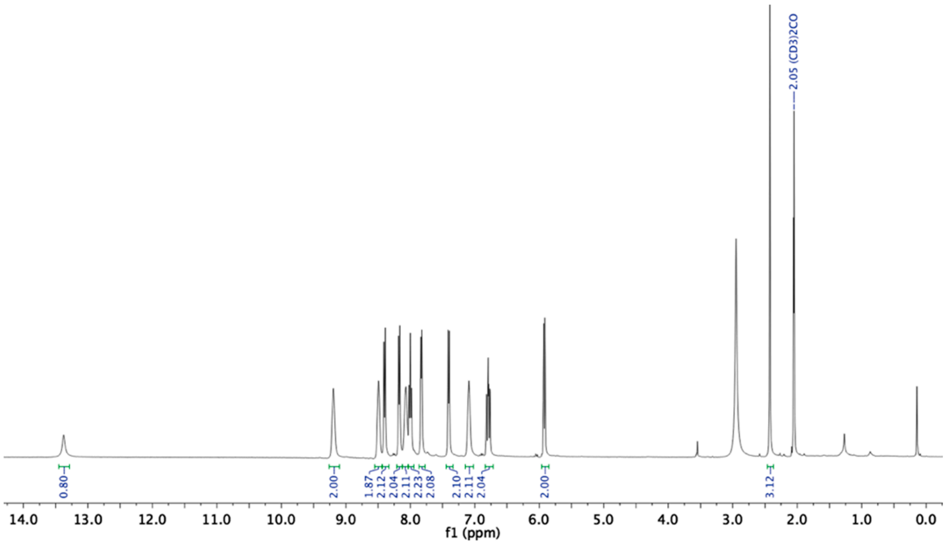

Figure S12. ^{1}H NMR (400 MHz, Acetone-d_6, 298 K)

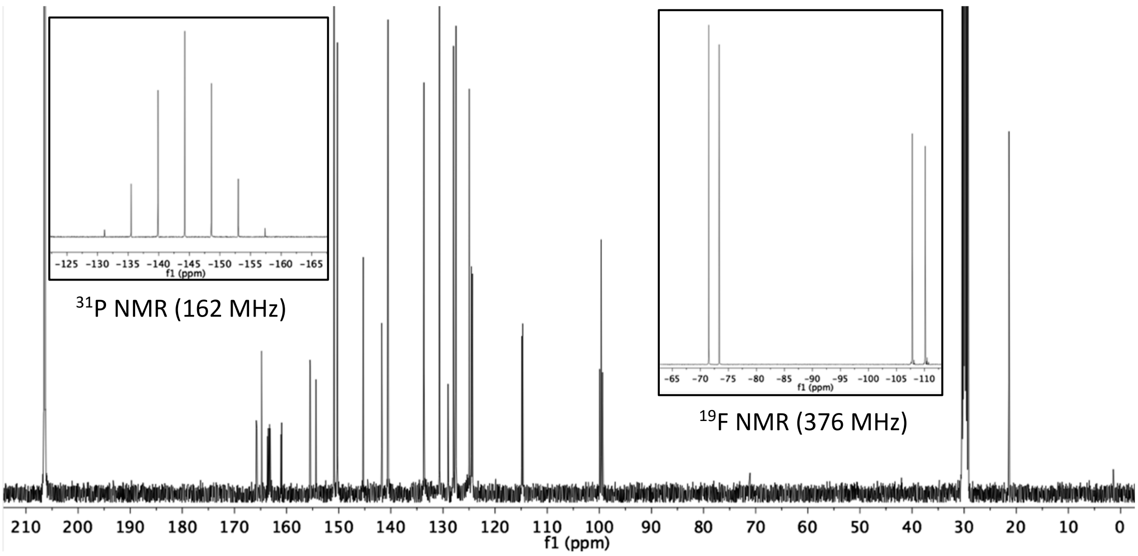

Figure S13. ^{13}C NMR (101 MHz, Acetone-d_6, 298 K) and inserts of ^{19}F NMR and ^{31}P NMR.

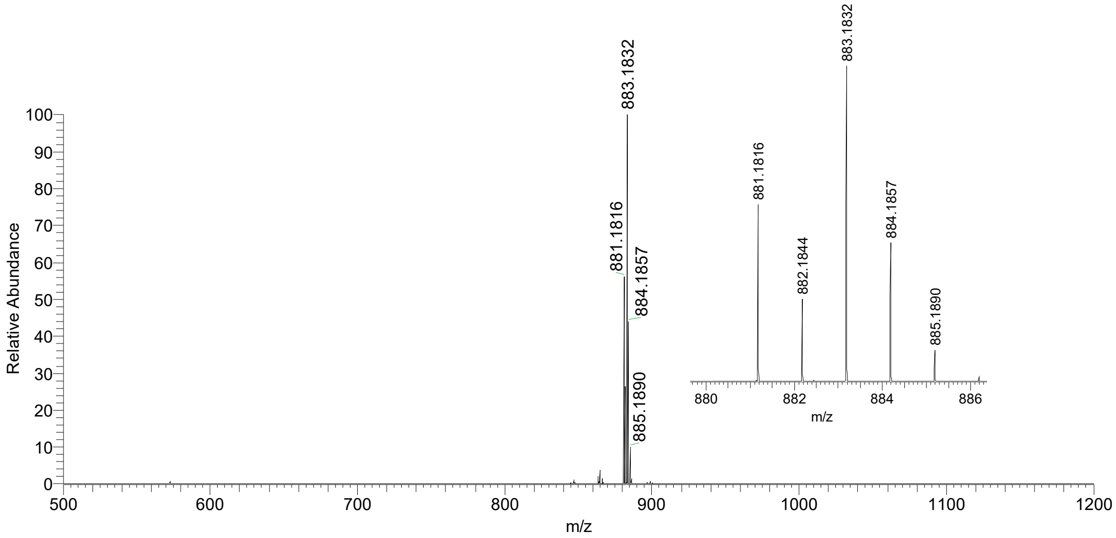

Figure S14. HRMS for **C5** (ESI – Positive Mode)

[(2-(4-fluorophenyl)-1*H*-imidazo[4,5-*f*][1,10]phenanthroline)bis(2-(2,4-difluorophenyl) pyridine)Ir(III)]PF_6 (**C6**) This complex was obtained using the same method as above, but using [Ir(F_2ppy)$_2$(μ-Cl)$_2$]) and **L3**

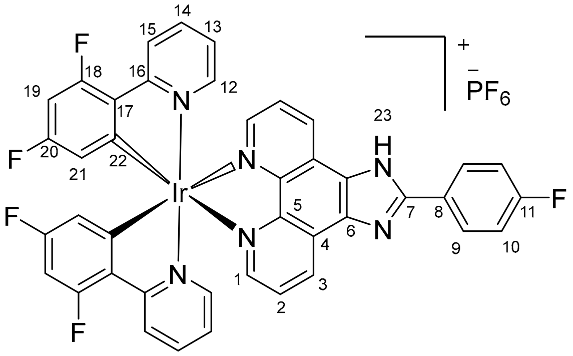

Yellow solid, 70 % yield.

^{1}H NMR (400 MHz, Acetone-d_6, 298 K) δ 9.16 (dd, *J* = 8.3, 1.4 Hz, 2H, H1), 8.48 (dd, *J* = 5.0, 1.2 Hz, 2H, H3), 8.42 (dt, *J* = 8.3, 1.6 Hz, 2H, H12), 8.16 (dd, *J* = 6.8, 3.0 Hz, 2H, H9), 8.05 – 7.97 (m, 4H, H2, H13), 7.86 (dd, *J* = 5.8, 1.4 Hz, 2H, H18), 7.65 – 7.40 (m, 3H, H10), 7.13 (ddd, *J* = 7.4, 5.8, 1.4 Hz, 2H, H14), 6.81 (ddd, *J* = 12.2, 9.4, 2.4 Hz, 2H, H19), 5.92 (dd, *J* = 8.5, 2.4 Hz, 2H, H21).

^{13}C NMR (101 MHz, Acetone-d_6, 298 K) δ 164.78 (d, $J^{C\text{-}F}$ = 7.1 Hz, C16), 164.49 (dd, $J^{C\text{-}F}$ = 256.0, 12.6 Hz, C20), 162.31 (d, $J^{C\text{-}F}$ = 260.1, 12.6 Hz, C18), 155.49 (C6a), 155.43 (C6b), 154.01 (C7), 150.92 (C12), 150.32 (C1), 145.40 (C5), 140.58 (C14), 133.78 (C3), 131.38 (C8), 130.18 (broad, C11), 130.02 (C10), 129.00 (d, $J^{C\text{-}F}$ = 4.5 Hz, C17), 127.98 (C2), 127.55 (C9), 125.39 (C4), 124.97 (C13), 124.45 (d, $J^{C\text{-}F}$ = 19.9 Hz, C15), 114.78 (dd, $J^{C\text{-}F}$ = 17.6, 2.9 Hz, C21), 99.68 (t, $J^{C\text{-}F}$ = 27.0 Hz, C19).

^{31}P NMR (162 MHz, Acetone-d_6, 298 K) δ -146.45 (hept, $J^{P\text{-}F}$ = 708.1 Hz, PF_6).

^{19}F NMR (376 MHz, Acetone-d_6, 298 K) δ -72.52 (d, $J^{F\text{-}P}$ = 707.1 Hz, PF_6), -107.83 (d, $J^{F\text{-}F}$ = 10.7 Hz, *p*-F-ppy), -110.12 (d, $J^{F\text{-}F}$ = 10.8 Hz, *o*-F-ppy), -150.74 (s, F-Ph).

IR (KBr): ν/cm^{-1} = 3367 (N-H stretching), 1523 (C=C stretching), 1365 (C-C stretching), 860 (P-F bending).

HRMS (ESI): m/z $[M]^+$ for $C_{41}H_{23}F_5IrN_6$: calc: 887,1534; found: 887,1574.

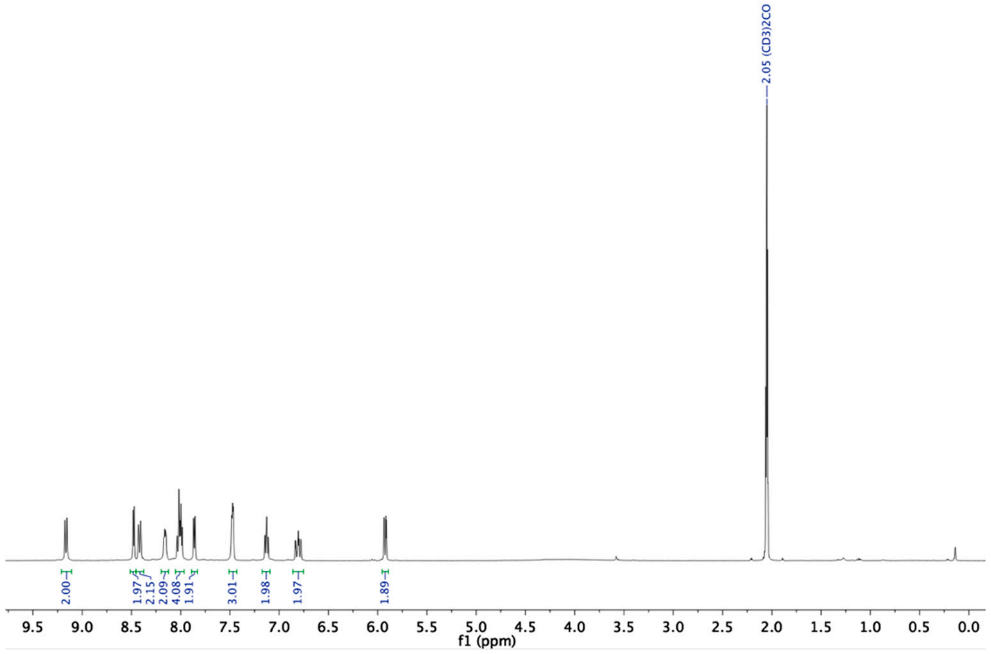

Figure S15. ^{1}H NMR (400 MHz, Acetone-d_6, 298 K)

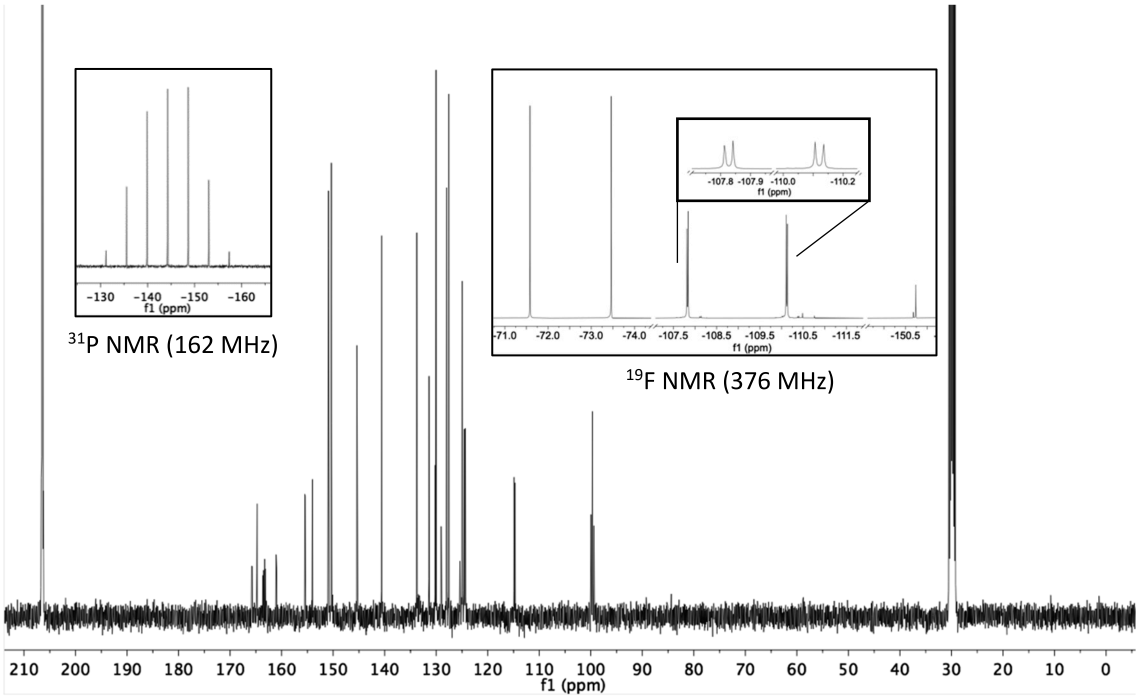

Figure S16. ^{13}C NMR (101 MHz, Acetone-d_6, 298 K) and inserts of ^{19}F NMR and ^{31}P NMR.

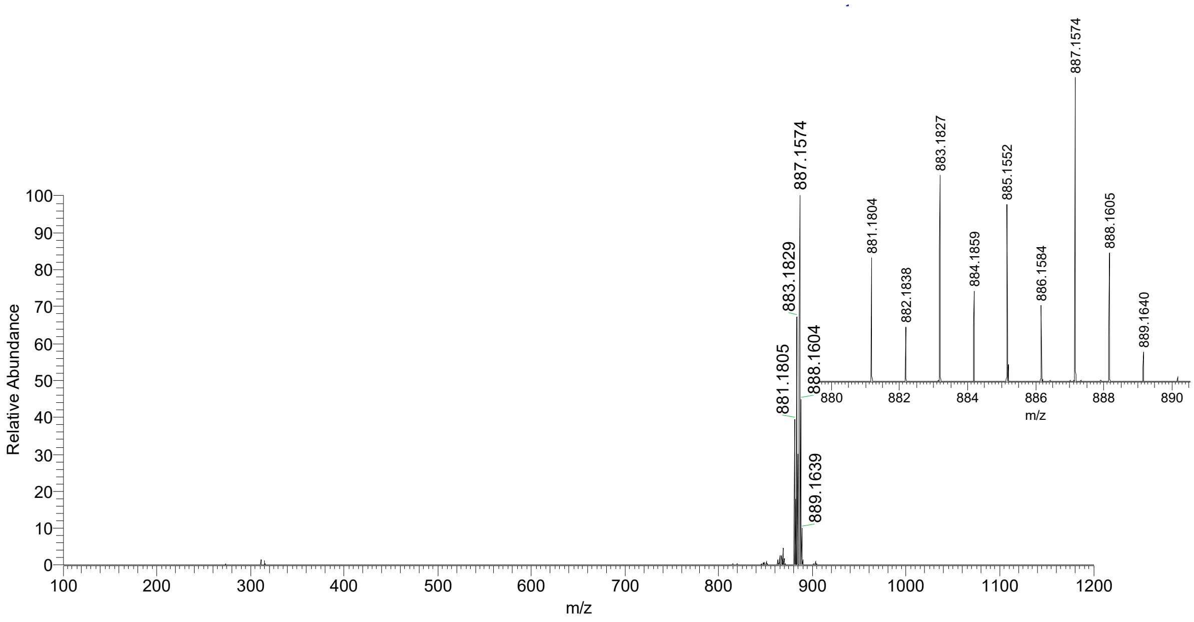

Figure S17. HRMS for **C6** (ESI – Positive Mode)

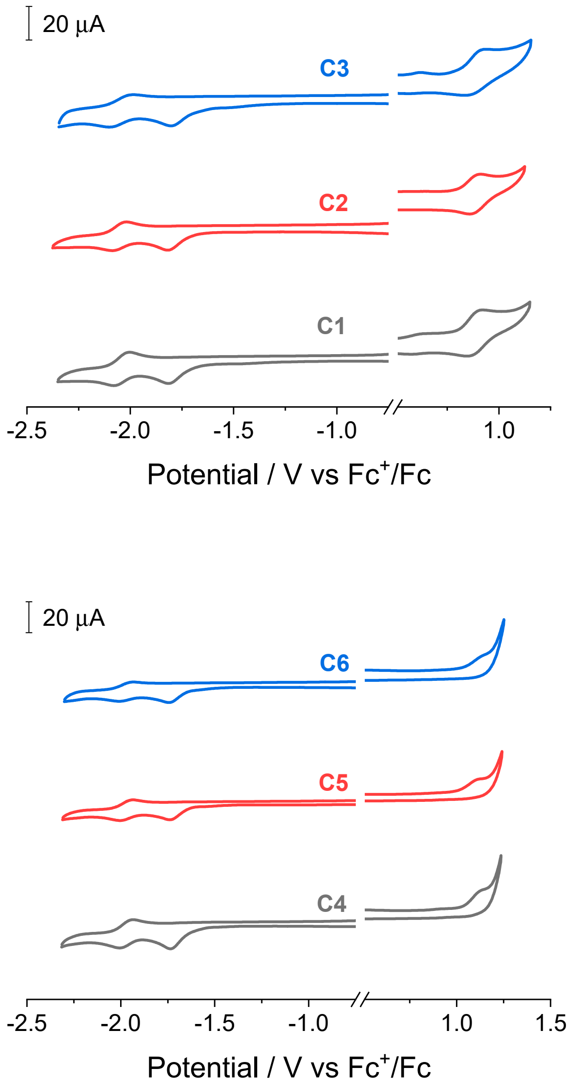

Figure S18. Cyclic voltammetry of complexes **C1-3** (top) and complexes **C4-6** (bottom) measured in acetonitrile (0.1 M $TBAPF_6$) at a scan rate of v = 0.1 V/s.

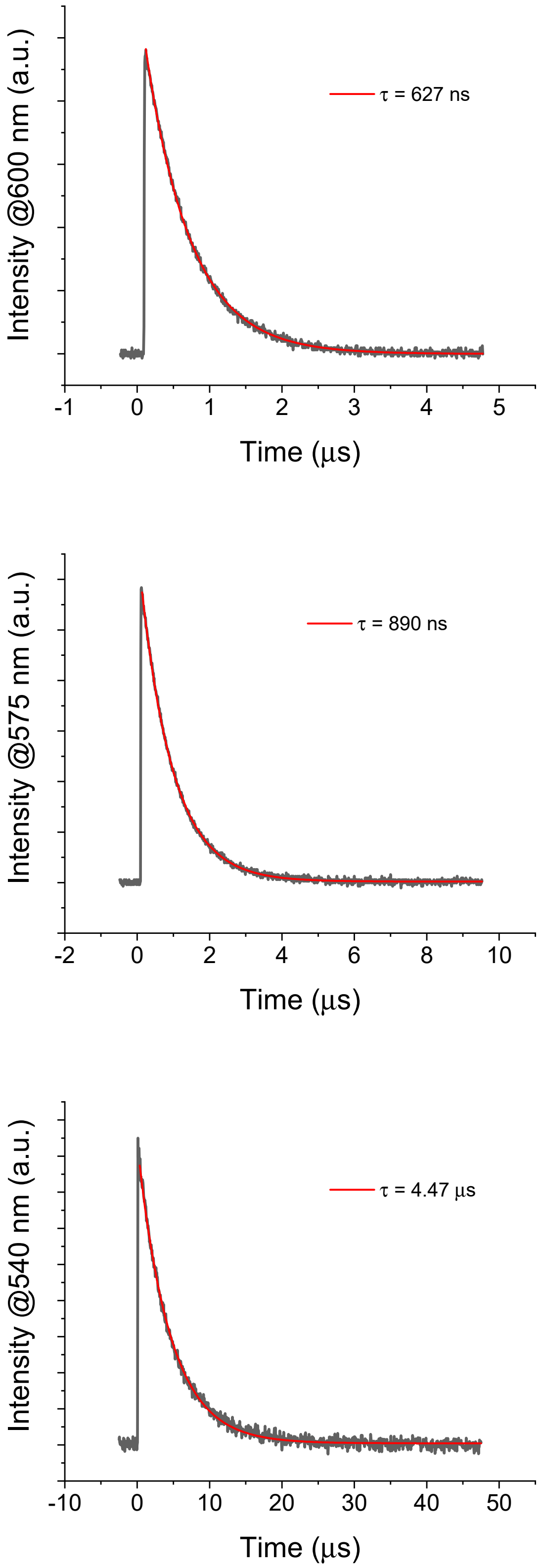

Figure S19. Time-resolved emission decays measured by laser flash photolysis (excitation at 355 nm) of **C1** in acetonitrile (top), dichloromethane (middle), and in a glassy matrix at 77 K (bottom).

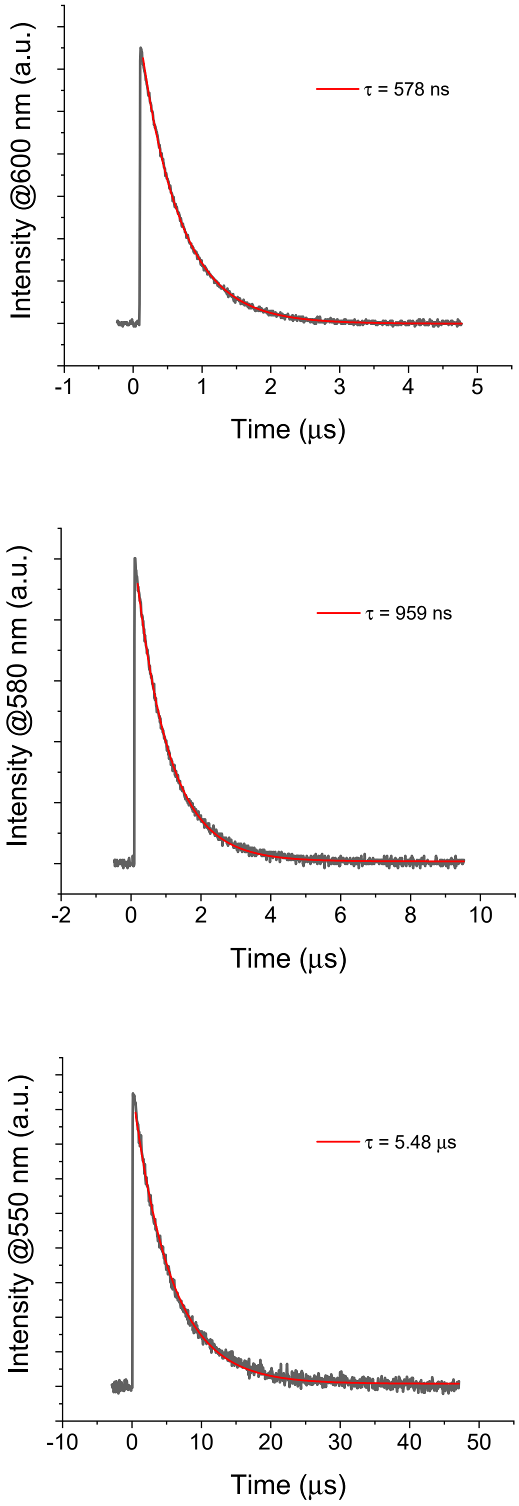

Figure S20. Time-resolved emission decays measured by laser flash photolysis (excitation at 355 nm) of **C2** in acetonitrile (top), dichloromethane (middle), and in a glassy matrix at 77 K (bottom).

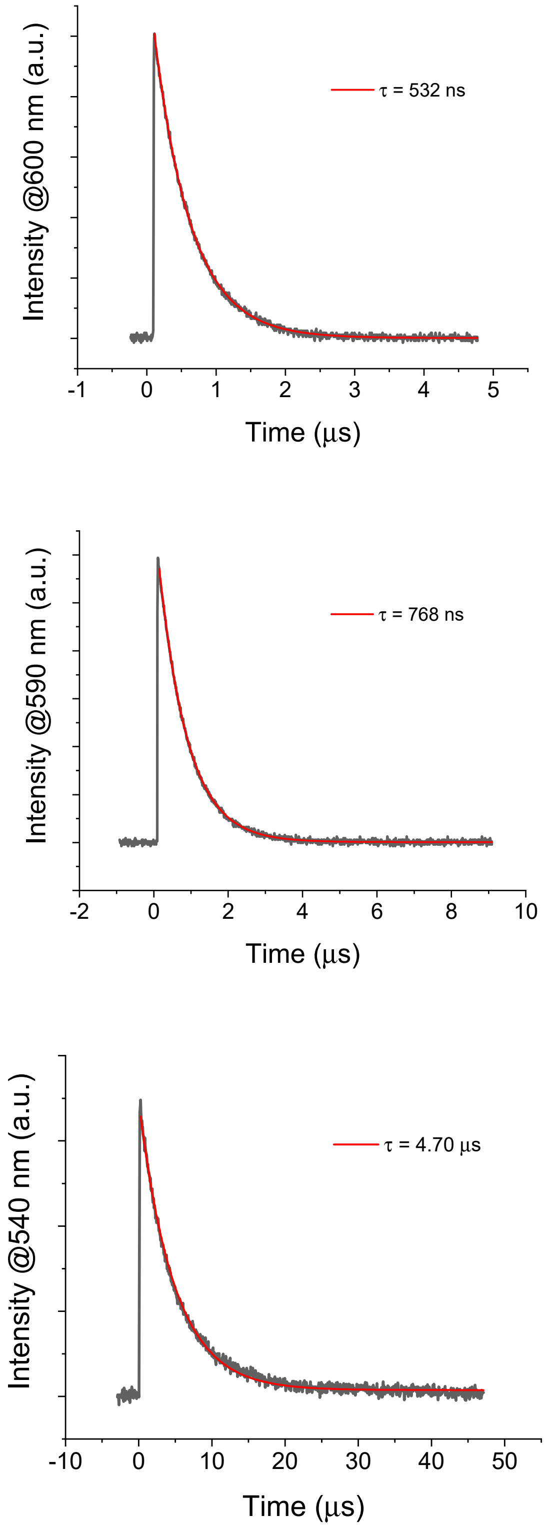

Figure S21. Time-resolved emission decays measured by laser flash photolysis (excitation at 355 nm) of **C3** in acetonitrile (top), dichloromethane (middle), and in a glassy matrix at 77 K (bottom).

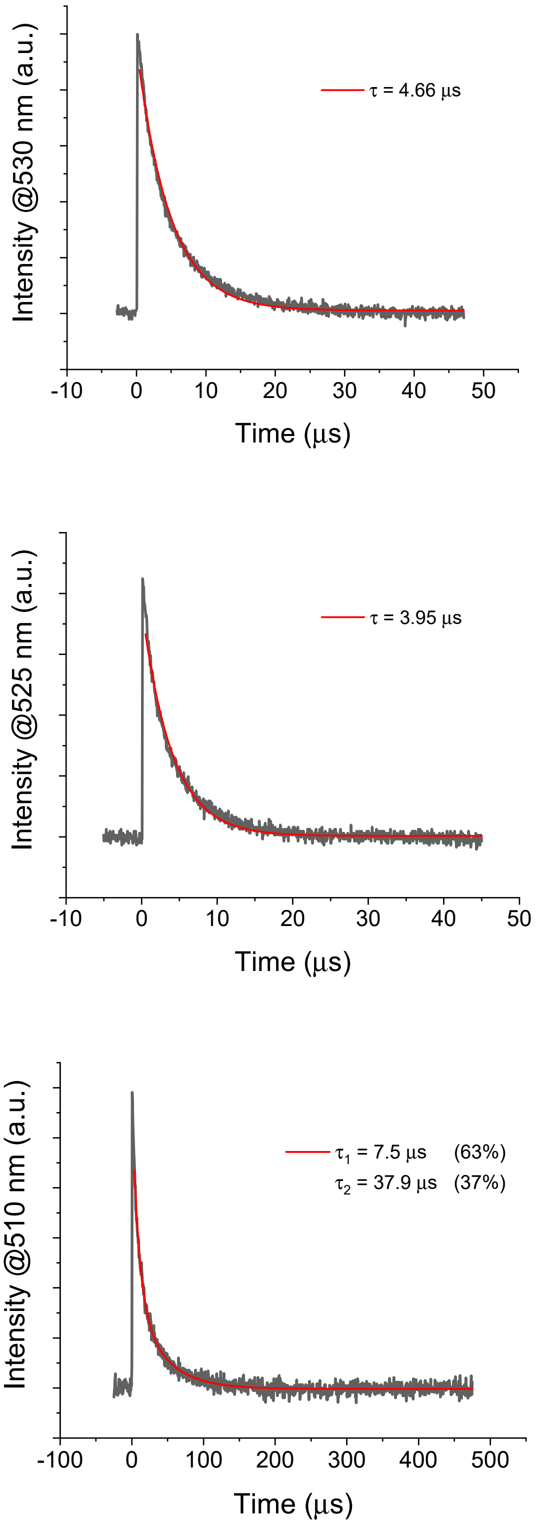

Figure S22. Time-resolved emission decays measured by laser flash photolysis (excitation at 355 nm) of **C4** in acetonitrile (top), dichloromethane (middle), and in a glassy matrix at 77 K (bottom).

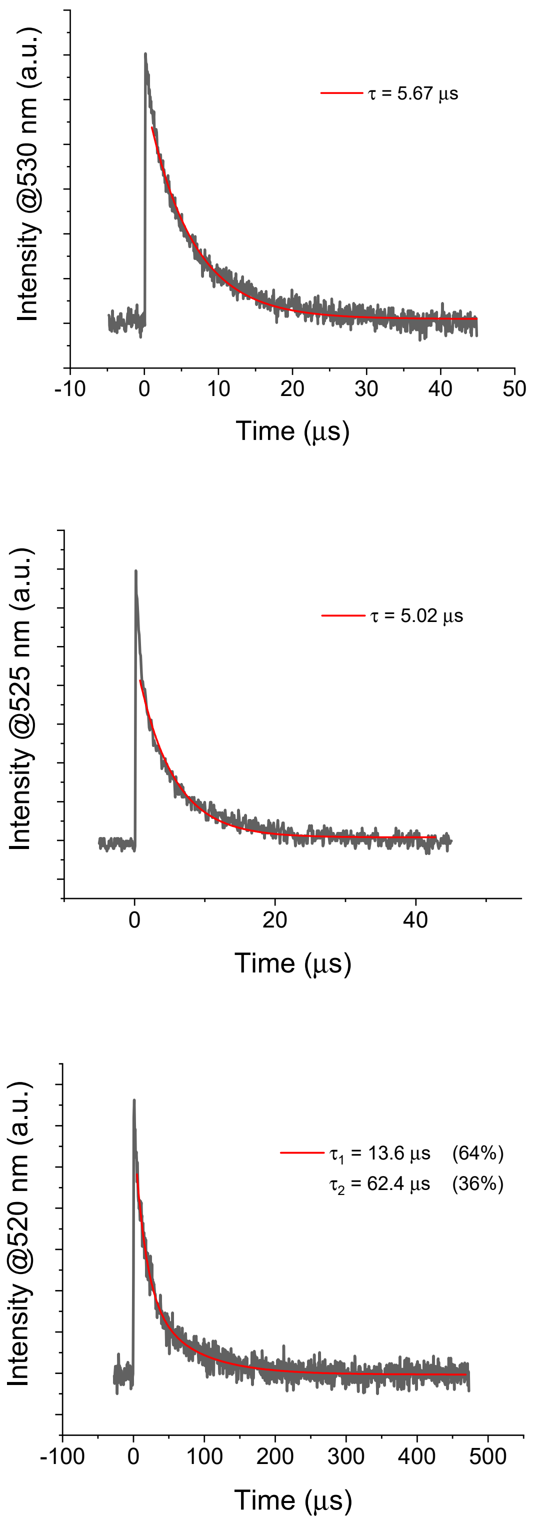

Figure S23. Time-resolved emission decays measured by laser flash photolysis (excitation at 355 nm) of **C5** in acetonitrile (top), dichloromethane (middle), and in a glassy matrix at 77 K (bottom).

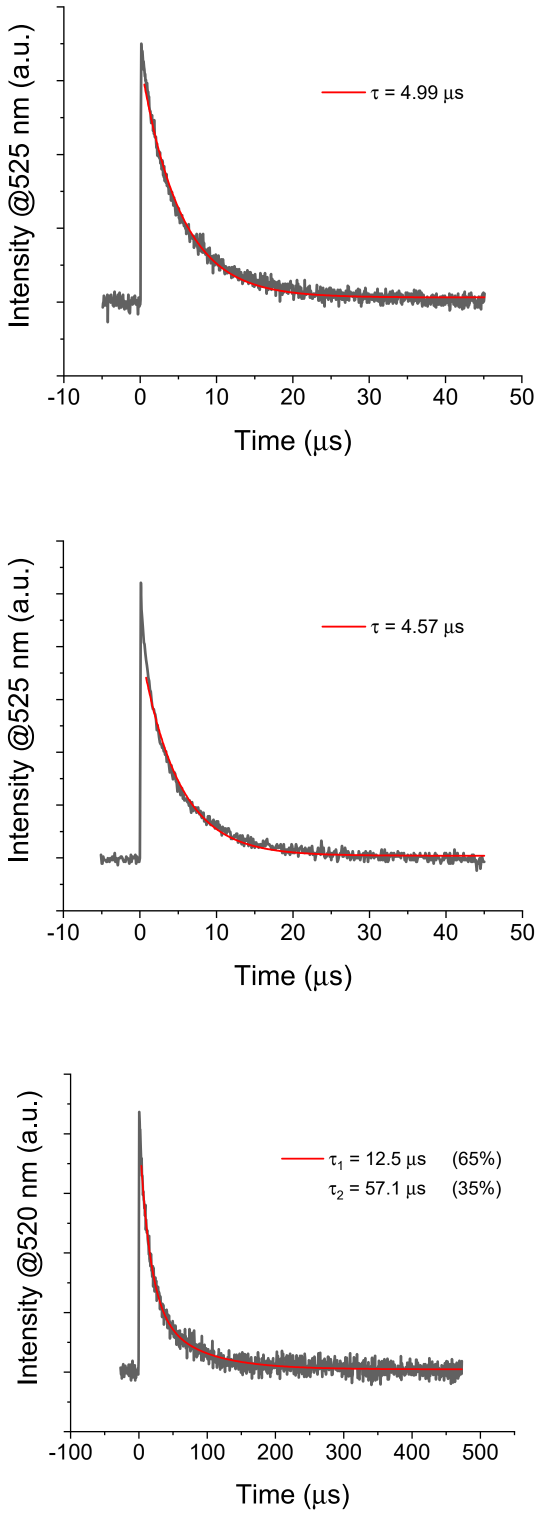

Figure S24. Time-resolved emission decays measured by laser flash photolysis (excitation at 355 nm) of **C6** in acetonitrile (top), dichloromethane (middle), and in a glassy matrix at 77 K (bottom).

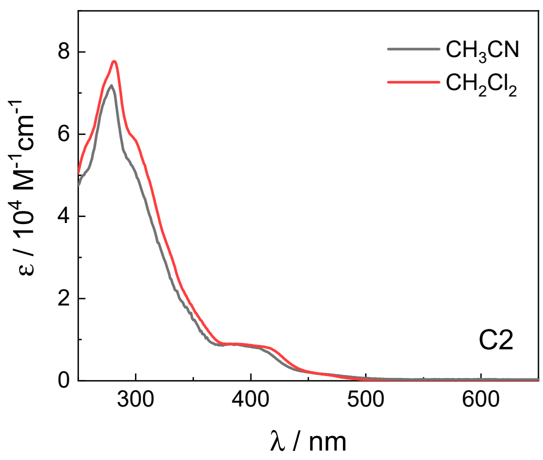

Figure S25. Absorption spectra of C2 complex in acetonitrile (black) and dichloromethane (red)

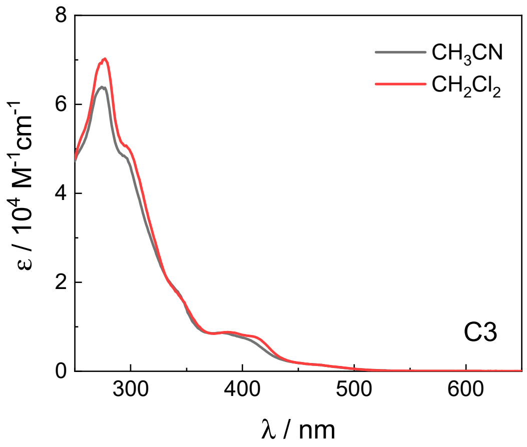

Figure S26. Absorption spectra of C3 complex in acetonitrile (black) and dichloromethane (red)

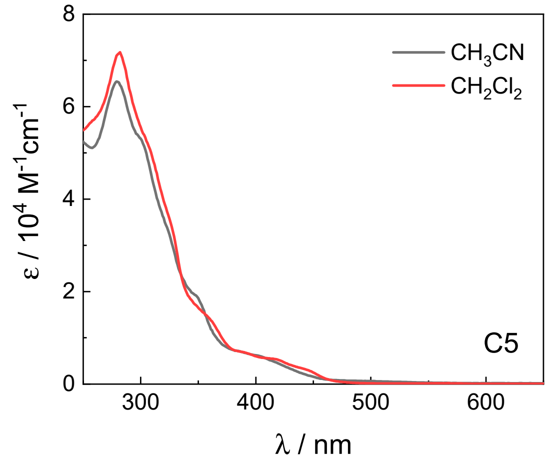

Figure S27. Absorption spectra of C5 complex in acetonitrile (black) and dichloromethane (red)

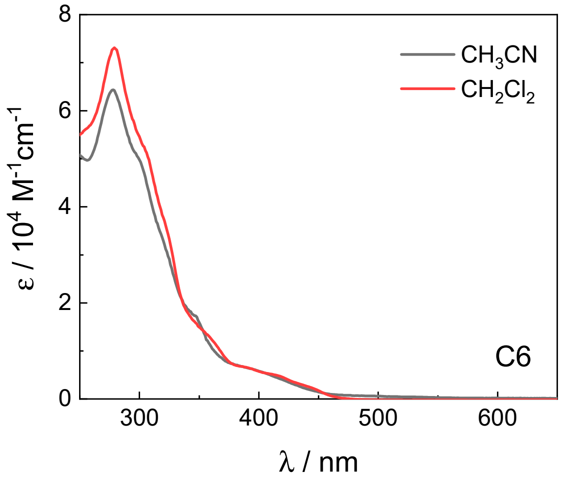

Figure S28. Absorption spectra of C6 complex in acetonitrile (black) and dichloromethane (red)
